# GADD45A suppression contributes to cardiac remodeling by promoting inflammation, fibrosis and hypertrophy

**DOI:** 10.1007/s00018-025-05704-x

**Published:** 2025-04-30

**Authors:** Adel Rostami, Xavier Palomer, Javier Pizarro-Delgado, Lucía Peña, Mònica Zamora, Marta Montori-Grau, Emma Barroso, Brenda Valenzuela-Alcaraz, Fàtima Crispi, Jesús M. Salvador, Raquel García, María A. Hurlé, Francisco Nistal, Manuel Vázquez-Carrera

**Affiliations:** 1 Department of Pharmacology, Toxicology and Therapeutic Chemistry, Faculty of Pharmacy and Food Sciences, University of Barcelona, Barcelona, 08028 España; 2 Institute of Biomedicine of the University of Barcelona (IBUB), University of Barcelona, Barcelona, 08028 Spain; 3 Spanish Biomedical Research Center in Diabetes and Associated Metabolic Diseases (CIBERDEM), Instituto de Salud Carlos III, Madrid, 28029 Spain; 4 Pediatric Research Institute, Hospital Sant Joan de Déu, Esplugues de Llobregat, 08950 Spain; 5 BCNatal - Fetal Medicine Research Center (Hospital Clínic and Hospital Sant Joan de Déu), University of Barcelona, Barcelona, 08028 Spain; 6 Institut d’Investigacions Biomèdiques August Pi i Sunyer (IDIBAPS), University of Barcelona, Barcelona, 08036 Spain; 7 Centre for Biomedical Research on Rare Diseases (CIBER-ER), Instituto de Salud Carlos III, Madrid, 28029 Spain; 8 Department of Immunology and Oncology, National Center for Biotechnology/CSIC, Madrid, 28049 Spain; 9 Departamento de Fisiología y Farmacología, Facultad de Medicina, Universidad de Cantabria, Instituto de Investigación Marqués de Valdecilla (IDIVAL), Santander, Spain; 10 Servicio de Cirugía Cardiovascular, Departamento de Ciencias Médicas y Quirúrgicas, Facultad de Medicina, Hospital Universitario Marqués de Valdecilla, Instituto de Investigación Marqués de Valdecilla (IDIVAL), Universidad de Cantabria, Santander, Spain; 11 Spanish Biomedical Research Center in Cardiovascular Diseases (CIBERCV), Instituto de Salud Carlos III, Santander, Spain

**Keywords:** Apoptosis, Cardiac hypertrophy, GADD45A, Inflammation and fibrosis

## Abstract

The growth arrest and DNA damage inducible 45A (GADD45A) is a multifaceted protein associated with stress signaling and cellular injury. Aside its well-established tumor suppressor activity, recent studies point to additional roles for GADD45A, including the regulation of catabolic and anabolic pathways, or the prevention of inflammation, fibrosis, and oxidative stress in some tissues and organs. However, little is known about its function in cardiac disease. In this study, we aimed to evaluate the role of GADD45A in the heart by using mice with constitutive and systemic deletion of *Gadd45a*, and cardiac cells of human origin. *Gadd45a* suppression in knockout mice triggered cardiac fibrosis, inflammation, and apoptosis, and these changes correlated with an hyperactivation of the pro-inflammatory and pro-fibrotic transcription factors activator protein-1 (AP-1), nuclear factor-κB (NF-κB), and signal transducer and activator of transcription 3 (STAT3). Deletion of *Gadd45a* also resulted in substantial cardiac hypertrophy, which negatively impacted cardiac morphology and function in knockout mice. Consistent with this, GADD45A overexpression in human AC16 cardiomyocytes partially prevented the inflammatory and fibrotic responses induced by tumor necrosis factor-α (TNF-α). Overall, data presented in this study highlight an important role for GADD45A in the heart, since it may prevent inflammation, fibrosis, and apoptosis, and, by this means, preserve cardiac function and performance. Since fibrosis and inflammation are crucial in the progression of cardiac hypertrophy and subsequent heart failure, these results suggest that promoting the activity of this protein might be a promising therapeutic strategy to slow down the progression of these deleterious diseases.

**Supplementary Information:**

The online version contains supplementary material available at 10.1007/s00018-025-05704-x.

## Introduction

The growth arrest and DNA damage inducible (GADD)45 family consists of three stress-responsive proteins named GADD45A (also known as GADD45α or DNA damage-inducible transcript [DDIT]1 protein), GADD45B (GADD45β or myeloid differentiation primary response 118, MYD118), and GADD45G (GADD45γ, DDIT2, or cytokine-responsive protein 6, CR6). The first member described was GADD45A, which is expressed in the brain, heart, kidney, liver, skeletal muscle, spleen, and lung. Its gene expression may be induced by a myriad of genotoxic and non-genotoxic stresses, and is tightly regulated by the combined action of transcriptional, post-transcriptional, and post-translational mechanisms that are dependent on the causative stressor and the cell type [1–5]. Historically, *GADD45A* expression has been considered primarily as a downstream event of the activation of the transcription factor p53, a widely recognized tumor suppressor [6]. GADD45A itself is regarded as a tumor suppressor capable of delaying, or even preventing, tumor development through the regulation of cell cycle, DNA repair, and apoptosis [7]. Consequently, mice lacking GADD45A exhibit genomic instability and reduced apoptosis of malignant cells, and, thus, are more prone to tumor development [6, 8–11]. In agreement with these valuable effects, GADD45A is reduced in many human tumors, and its gene expression positively correlates with a higher survival rate and a better prognosis in patients with cancer [12, 13]. In addition to p53, other regulatory factors may transcriptionally regulate *GADD45A*, either directly or indirectly, including activating transcription factor 4 (ATF4), early growth response protein 1 (EGR1), nuclear transcription factor Y subunit α (NFYA), POU domain class 2 transcription factor 1 (POU2F1), Wilms tumor protein 1 (WT1), and even the pro-inflammatory and pro-fibrotic regulators activator protein-1 (AP-1), nuclear factor-κB (NF-κB), and nuclear factor of activated T-cells (NFAT) [3, 7, 12]. Actually, NF-κB and AP-1 are themselves involved in the regulation of cell differentiation, proliferation, and apoptosis in response to diverse stimuli (cytokines, growth factors, stress, and bacterial or viral infections) [5]. On the other hand, a very recent study has demonstrated a role for GADD45A in maintaining intestinal barrier integrity and the development of ulcerative colitis through its downstream Wnt/β-catenin pathway, which regulates cell proliferation, differentiation, and homeostasis of intestinal epithelia [14].

Beyond these well-known effects, other roles for GADD45A have been reported in recent years. Studies using knockout (KO) mice have demonstrated that this protein controls the immune response during autoimmunity [15], whereas its overexpression in animal models prevents hepatic inflammation, fibrosis, apoptosis, oxidative stress and endoplasmic reticulum (ER) stress, while it improves fatty acid (FA) metabolism [16]. GADD45A has also been associated with resistance to ferroptosis through the activation of antioxidant pathways [17]. In adipose tissue, GADD45A regulates adipogenesis, lipid accumulation, and thermogenic metabolism, thus improving insulin sensitivity, glucose uptake and energy expenditure [18]. In this regard, some authors have pointed out that GADD45A modulation might be a suitable therapeutic approach to prevent obesity and diabetes. For example, a recent study demonstrates that GADD45A deficiency protects mice against high-fat diet-induced obesity [19]. The regulation of genes involved in metabolism, mitochondrial function, autophagy, and proteolysis, also accounts for the pivotal role that GADD45A exerts during muscle atrophy [20]. GADD45A has also been recently described as a critical regulator of intramuscular fat infiltration, a common feature during ageing, obesity and myopathies associated with muscular dysfunction and sarcopenia [21].

Cardiac hypertrophy is an adaptive response that develops because of increased workload, and which aims to reduce ventricular wall stress while maintaining heart function and efficiency. Physiological hypertrophy is a fully reversible and adaptive process that does not result in structural or functional cardiac abnormalities, since it occurs in the absence of inflammation, fibrosis or cell death [22]. However, if the causative stimulus persists, it may progress to pathological hypertrophy, characterized by ventricular chamber dilation, contractile dysfunction and, finally, heart failure, arrhythmias, and death [22–24]. The main mechanisms of pathological hypertrophy entail inflammation, fibrosis, mitochondrial dysfunction, dysregulation of calcium-handling proteins, metabolic changes, fetal gene expression reactivation, cardiomyocyte hypertrophy, and cell death [22, 24, 25]. Fibrosis, in particular, directly correlates with disease progression and adverse clinical outcomes, and has a great impact on the clinical condition of the patient [26]. Of note, although GADD45A has been somehow associated with hypertrophic [27] and diabetic cardiomyopathies [28], little is known about its function in the heart. Therefore, the aim of this study consisted in evaluating the role of GADD45A in the heart by using knockout mice and cardiac cells of human origin.

## Materials and methods

### Human samples

This study was carried out using left ventricular myocardial intraoperative biopsies obtained from a cohort of 59 patients (31 women) who were diagnosed with isolated severe aortic stenosis and underwent aortic valve replacement surgery in the University Hospital Marqués de Valdecilla in Santander (Spain). Patients with aortic or mitral regurgitation greater than mild or with major coronary stenosis > 50%, previous cardiac operations, malignancies, or poor renal or hepatic function were deemed ineligible for the study. The control group comprised a cohort of 30 surgical patients (17 women) with pathologies not associated with left ventricle (LV) pressure or volume overload, coronary heart disease, or cardiomyopathies. The demographic and clinical characteristics of the patients included in this study are shown in Supplementary Table S3. Subepicardial biopsies (40 mg) were taken from the left ventricle lateral wall with a Tru-cut needle during the surgical procedure. Samples were all harvested by the same surgeon in a protocolized manner and always from the same location in the margo obtusus of the heart. The study followed the Declaration of Helsinki guidelines for biomedical research involving human subjects. The Institutional Ethics and Clinical Research Committee of Cantabria approved the study (internal authorization code PI21/00084). All patients were informed verbally and through printed material about the nature of the study and provided written informed consent.

### Transthoracic echocardiography of human patients

During the week prior to cardiac surgery, patients with either a pure severe aortic valve stenosis (experimental group) or patients with pathologies not producing LV pressure or volume overload (control group), underwent a transthoracic two-dimensional echocardiographic study (Philips-Hewlett Packard, IE33™ or Epiq 7™, Amsterdam, Netherlands). Images were digitalized and analyzed off-line (Xcelera software™, Philips, Amsterdam, Netherlands). Left ventricular chamber dimensions and wall thicknesses were measured according to the American Society of Echocardiography guidelines with bidimensional or M-mode images. Mitral annular plane systolic excursion (MAPSE) was assessed in the septal mitral annulus by 2D-guided M-mode echocardiography in the four-chamber view. LV ejection fraction (EF) was calculated with the Quiñones formula, and LV mass (LVM) was estimated according to the Devereux formula and indexed to patient height in meters to the 2.7th power. This method of normalization of LV mass accounts for biases related to sex or body habitus and is less influenced by obesity than the normalization by body surface area.

### Mouse cardiac sample preparation

Male mice with constitutive and systemic deletion of *Gadd45a* (*Gadd45a* KO) and their control wild-type (WT) littermates with the same genetic background (B6129F1) were used [15]. Mice were housed under standard light-dark cycle (12-h light/dark cycle, 300 lux/0 lux) and temperature (21 ± 1ºC) conditions, and food and water were provided *ad libitum*. The hearts were collected at 24 weeks of age from mice euthanized using deep isoflurane (5%) anesthesia, rinsed in ice-cold phosphate buffer saline, and snap-frozen in liquid nitrogen. For the glucose tolerance test, animals were fasted for 4 h before receiving 2 g/Kg body weight of glucose (#G7021, Sigma-Aldrich Corporation, St. Louis, MO, USA) by intraperitoneal injection. Next, blood was collected from the tail vein after 0, 20, 40 and 90 min. The pyruvate tolerance test was performed in animals fasted for 15 h. Then, each animal was injected 2 g/Kg of sodium pyruvate (#P2256, Sigma-Aldrich) intraperitoneally, and blood glucose samples were collected from the tail vein at 15, 30, 45, 60, 75, 90, 105 and 120 min. All animal procedures were performed in accordance with European Community Council directive 86/609/EEC and followed the ARRIVE and the standard ethical guidelines [29], and were approved by the Institutional Animal Care and Use Committee of the University of Barcelona, as stated in Law 5/21 July 1995 passed by the Generalitat de Catalunya. Accordingly, all efforts were made to minimize the suffering and the number of mice used.

### Transthoracic echocardiography of mice

Mice were sedated using a mixture of isoflurane (5%) and oxygen (2 L/min), which was subsequently reduced to 1.5% isoflurane to maintain the heart rate in the range of 350–450 beats/min. Transthoracic echocardiography was performed from a longitudinal parasternal plane using a Vivid Q (GE Healthcare, Norway) echocardiograph equipped with a 5.0–13.0 MHz 33 mm linear probe. The acquired images’ offline analysis was used to determine EF, fractional shortening (FS), septal and LV free wall thicknesses, and end diastolic and end systolic diameters by M-mode.

### Histology

Hearts were fixed in 4% buffered paraformaldehyde and paraffin-embedded for subsequent hematoxylin and eosin or Masson’s trichrome stains. Myocardial transverse sections (5 μm thick) were visualized using an Olympus BX41 microscope equipped with a DP11 CCD camera (Olympus Iberia, Barcelona, Spain), and digital images were obtained at 200X magnification. The cardiomyocyte cross-sectional area was analyzed in approximately 50 cells of two randomly chosen sections per mouse using ImageJ software (National Institutes of Health, USA). The degree of fibrosis was determined in two randomly chosen frames from Masson’s trichrome-stained sections, and the area of fibrosis was determined using ImageJ software. The percentage of interstitial fibrotic areas was calculated as the fraction of the light-blue–stained area×100%.

### Cell culture and transfection

The human AC16 cell line, which develops many of the biochemical and morphological properties characteristic of cardiac muscle cells, even though it does not form completely differentiated cardiomyocytes, was grown as previously described [30]. Briefly, non-differentiated AC16 cells (Merck Millipore, Burlington, MA, USA) were maintained in medium composed of Dulbecco’s modified Eagle’s medium (DMEM) (Life Technologies, Spain) supplemented with 10% fetal bovine serum (FBS), 1% penicillin-streptomycin and 1% Fungizone (Life Technologies), and grown at 37 °C in a humid atmosphere of 5% CO2/95% air until they reached 70–80% confluence. For overexpression studies, cells were transfected with the pCMV3-GADD45A (Sino Biological Inc., Eschborn, Germany) or the pcDNA3/LacZ plasmid as a control. Cells were transfected for 48 h with Lipofectamine 2000 in OPTI-MEM reduced serum medium following the manufacturer’s recommendations (Life Technologies). Transfection time and the DNA to Lipofectamine ratio were set after optimization with the corresponding *LacZ*-carrying plasmid and using a β-galactosidase reporter gene staining kit (Sigma-Aldrich). Tumor necrosis factor-α (TNF-α; #H8916, Sigma-Aldrich) was added to the culture medium at a concentration of 10 ng/mL for the last 24 h before sample collection. Small interfering RNA (siRNA)-mediated *GADD45A* gene silencing was carried out by transfecting AC16 cells with human GADD45A siRNA (sc-35440; Santa Cruz Biotechnology, Inc., Dallas TX, USA), using scrambled siRNA as a transfection control. Fluorescein-labeled siRNA was used to optimize siRNA transfections by means of fluorescence microscopy.

### RNA preparation and analysis

Total RNA was isolated using Ultraspec reagent (Biotecx, Houston, TX, USA). RNA samples were cleaned (NucleoSpin RNA; Macherey-Nagel, Düren, Germany) and checked for integrity by agarose gel electrophoresis. The total RNA isolated by this method was undegraded and free of protein and DNA contamination. Relative levels of specific mRNAs were assessed by real-time RT-PCR, as previously described [31]. Reverse transcription was performed from 0.5 µg total RNA using Oligo(dT)_23_ and M-MLV Reverse Transcriptase (Life Technologies). The PCR reaction contained 10 ng of reverse-transcribed RNA, 2X IQ™ SYBRGreen Supermix (Bio-Rad, Barcelona, Spain) and 900 nM of each primer (for primer sequences see Supplementary Table S1). PCR assays were performed on a BioRad MiniOpticon™ Real-Time PCR system. Thermal cycling conditions were as follows: activation of Taq DNA polymerase at 95ºC for 10 min, followed by 40 cycles of amplification at 95ºC for 15 s and at 60ºC for 1 min. Optimal primer amplification efficiency for each primer set was assessed and a dissociation protocol was carried out to ensure a single PCR product.

### Immunoblot analysis

To obtain total protein extracts, AC16 cardiac cells or frozen tissue pieces were lysed in cold RIPA buffer containing phosphatase and protease inhibitors (5.4 µg/mL aprotinin, 20 µg/mL leupeptin, 0.2 mM phenylmethylsulfonyl fluoride, 1 mM sodium orthovanadate, 5 mM sodium floride). The homogenate was then centrifuged at 10,000×*g* for 30 min at 4 °C, and the supernatant protein concentration was determined using the Pierce BCA protein assay kit (Thermo Fisher Scientific, Waltham, MA, USA). For immunoblotting, protein fractions were separated by sodium dodecyl sulfate-polyacrylamide gel electrophoresis (SDS-PAGE) on 10% separation gels and transferred to Immobilon polyvinylidene difluoride membranes. Proteins were detected with several antibodies (Supplementary Table S2) using the Western Lightning^®^ Plus-ECL chemiluminescence kit (Thermo Fisher Scientific) and their size was estimated using protein molecular mass standards (Life Technologies).

### Statistical analysis

Statistical differences were established by either the Student’s t-test, Mann Whitney test, or one-way ANOVA, according to the number of groups compared. The parametric Pearson or non-parametric Spearman correlation coefficients were used to calculate the correlation for continuous variables using only complete pairs of observations. Before analysis, the normality and homoscedasticity of data distribution were checked by Shapiro-Wilk and Bartlett tests, respectively. Statistical analyses were performed with GraphPad Prism 10 software (GraphPad Software Inc., San Diego, CA, USA). Values of *p <* 0.05 were considered statistically significant.

## Results

### GADD45A negatively correlates with left ventricle mass and fibrosis markers in human samples

**Fig. 1:**
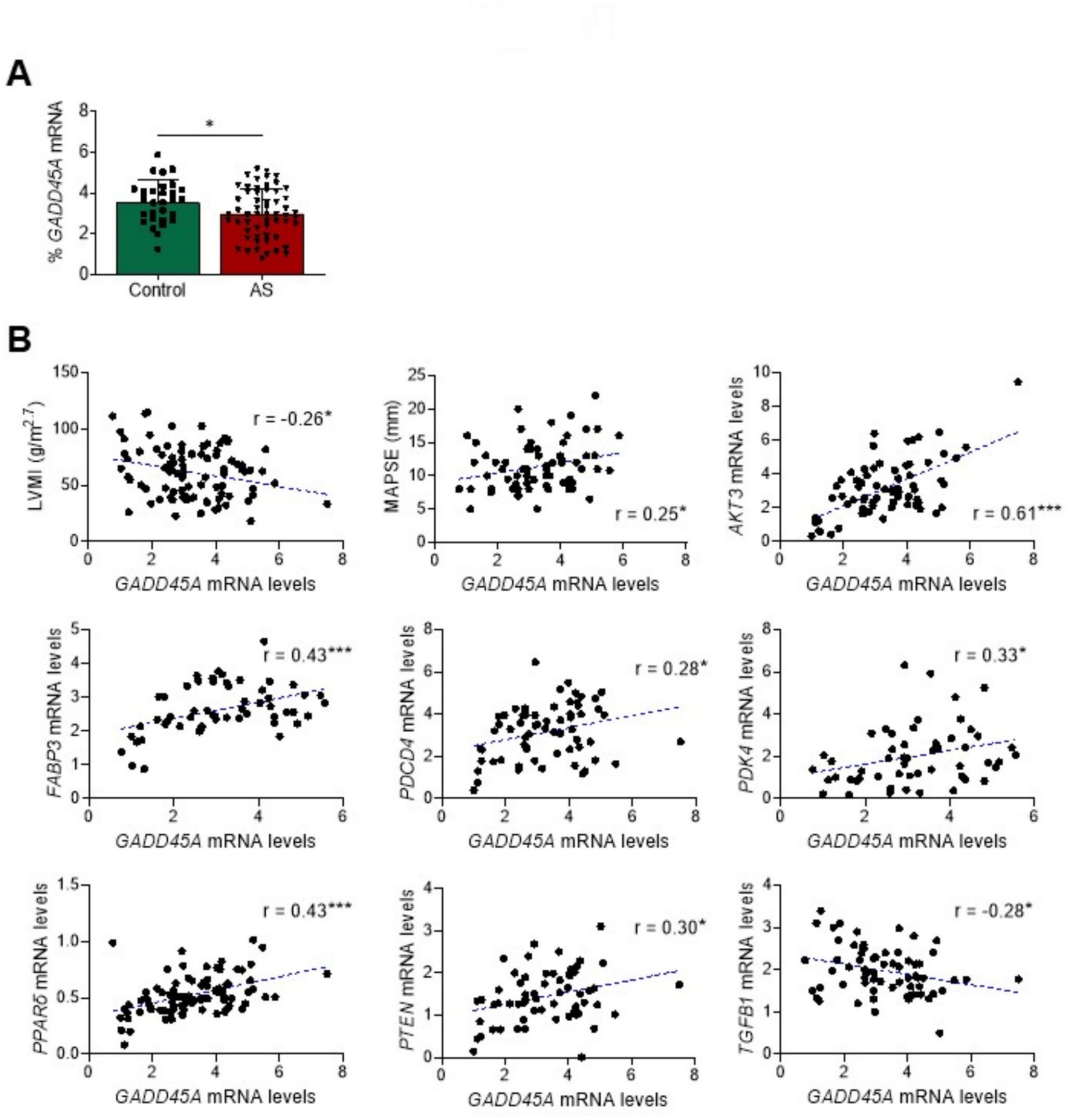
*GADD45A* gene expression negatively correlates with *TGFB1* in human samples. (**A**) Relative quantification of the mRNA expression of *GADD45A* in left ventricular myocardial intraoperative biopsies obtained at surgery in patients with severe aortic stenosis (AS) compared to control subjects. The graph represents the quantification of the glyceraldehyde-3-phosphate dehydrogenase (*GAPDH*)-normalized mRNA levels, expressed as a percentage of control samples. Data are presented as the mean ± SD. (**B**) Correlation coefficients between *GADD45A* mRNA levels and the indexed left ventricle mass (LVMI, normalized to the 2.7 power of height to neutralize differences in somatometry), mitral annular plane systolic excursion (MAPSE), and the mRNA levels of *AKT3*, *FABP3*, *PDK4*, *PDCD4*, *PPARD*, *PTEN*, and *TGFB1* in the same samples. The parametric Pearson or non-parametric Spearman correlation coefficients were used to calculate the correlation for continuous variables using only complete pairs of observations. The relative transcript levels of the target genes, in arbitrary units, were used to calculate these correlation coefficients. *p < 0.05, **p < 0.01, *** *p* < 0.001

Clinical data obtained from left ventricular myocardial intraoperative biopsies obtained at surgery showed lower levels of *GADD45A* expression in patients with aortic stenosis compared to control subjects (Fig. 1A). When analyzing all samples together, an inverse relationship (Pearson coefficient *r* = -0.26, *p* < 0.05, Fig. 1B) was observed between *GADD45A* mRNA levels and the LV mass indexed to the 2.7th power of the patient’s height. *GADD45A* positively correlated with MAPSE (Spearman coefficient *r* = 0.25, *p* < 0.05, Fig. 1B), which is associated with a lower risk for all-cause cardiac mortality, and also serves as a fair marker of structural abnormalities [32]. MAPSE is a surrogate of LV longitudinal strain and is both an early marker of ongoing systolic dysfunction and an indicator of myocardial fibrosis. The longitudinal orientation of subendocardial muscle fiber strata explains why their involvement in a fibrotic process translates in impaired long-axis contraction. The subendocardium is in turn the area most vulnerable to ischemia and fibrosis in the pressure overload situation that patients with aortic valve stenosis develop [33]. There was also a significant inverse correlation of *GADD45A* mRNA levels with the expression of transforming growth factor β1 (*TGFB1*, Pearson coefficient *r* = -0.28, *p* < 0.05), and a direct correlation with that of serine/threonine kinase *AKT3* (Pearson coefficient *r* = 0.61, *p* < 0.001), which promotes cardiac growth and mediates cardioprotective effects [34], FA binding protein 3 (*FABP3*, Pearson coefficient *r* = 0.43, *p* < 0.001), pyruvate dehydrogenase kinase 4 (*PDK4*, Pearson coefficient *r* = 0.35, *p* < 0.05), peroxisome proliferator-activated receptor (PPAR)β/δ (*PPARD*, Pearson coefficient *r* = 0.43, *p* < 0.001), and the tumor suppressor genes pro-apoptotic programmed cell death 4 (*PDCD4*, Pearson coefficient *r* = 0.28, *p* < 0.05) and phosphatase and tensin homolog (*PTEN*, Pearson coefficient *r* = 0.30, *p* < 0.05; Fig. 1B) [35, 36].

### GADD45A modulates inflammation and fibrosis in human cardiac AC16 cells

**Fig. 2:**
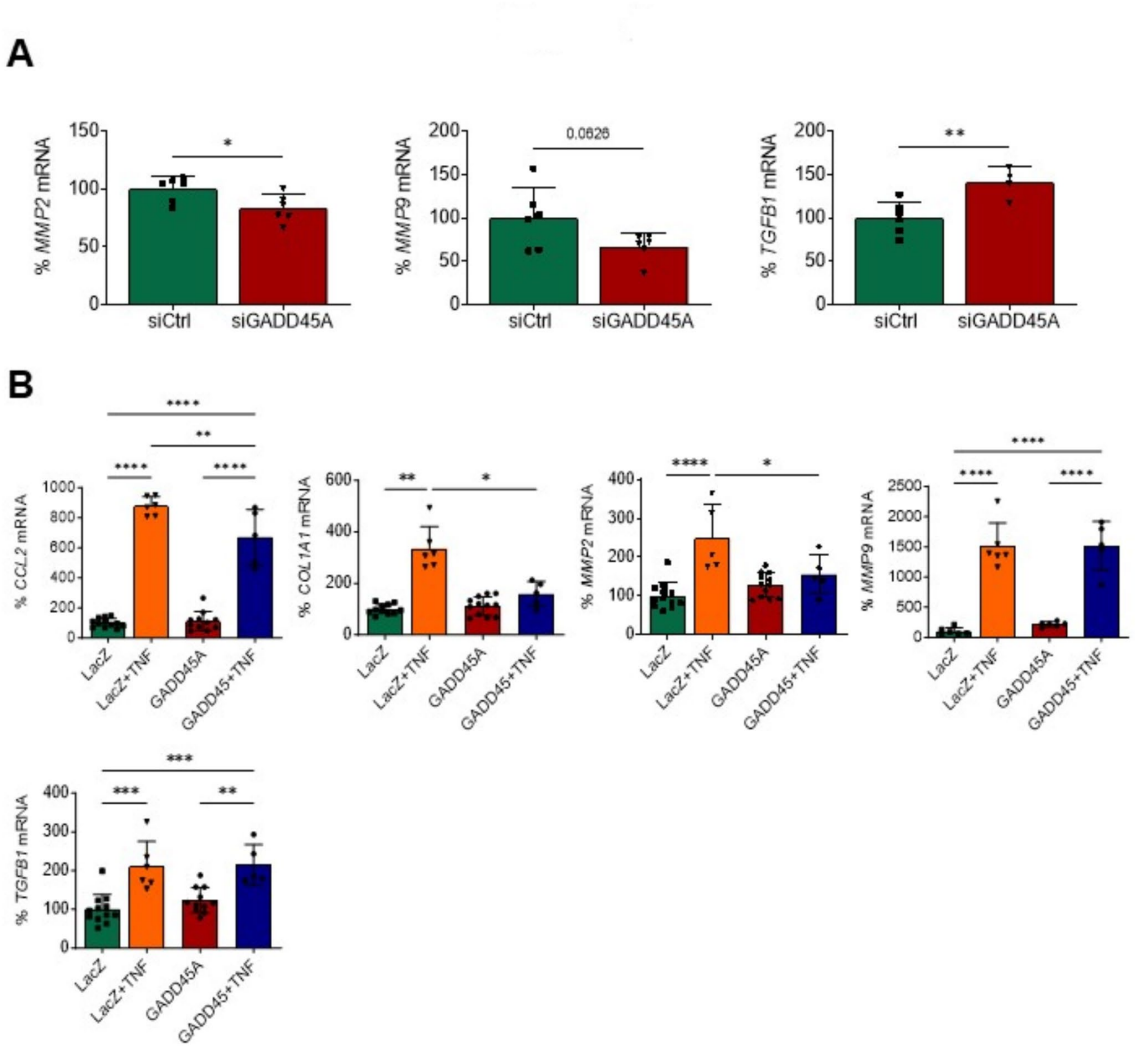
GADD45A prevents TNF-α-induced inflammation in human cardiac cells. (**A**) Relative quantification of the mRNA expression of *MMP2*, *MMP9*, and *TGFB1* in human AC16 cardiac cells transfected with scrambled siRNA (siRNA control, siCtrl), or GADD45A siRNA (siGADD45A). (**B**) Relative quantification of the mRNA expression of *CCL2*, *COL1A1*, *MMP2*, *MMP9*, and *TGFB1* in human AC16 cardiac cells transfected with LacZ-carrying or GADD45A-carrying plasmids in the presence or absence of TNF-α (TNF, 10 ng/mL, 24 h). The graphs represent the quantification of the glyceraldehyde-3-phosphate dehydrogenase (*GAPDH*)-normalized mRNA levels, expressed as a percentage of control (A, siCtrl; B, LacZ) samples. Data are presented as the mean ± SD. *p < 0.05, **p < 0.01, *** *p* < 0.001

To further confirm the important role of GADD45A in the human heart, *GADD45A* gene silencing was carried out by transfecting cultured cardiac cells of human origin (AC16) with human *GADD45A* siRNA. The reduction of up to 70% in *GADD45A* expression (Supplementary Fig. S1A) resulted in a reduction of matrix metallopeptidase (*MMP*)*2* and *MMP9* mRNA levels, and an increase in *TGFB1* expression as compared to control cells transfected with scrambled siRNA (Fig. 2A). However, downregulation of *GADD45A* was not a strong enough stimulus to induce a full pro-inflammatory profile in AC16 cells, since the gene expression of other pro-inflammatory markers was not significantly modified, including chemokine (C-C motif) ligand 2 (*CCL2*, also referred to as monocyte chemoattractant protein 1, *MCP1*), cellular communication network factor 2 (*CCN2*, or connective tissue growth factor, *CTGF*), interleukin 6 (*IL6*), or *TNF-α* (Supplementary Fig. S1B). Thus, we next explored the effects of *GADD45A* overexpression in these cells in the presence of a strong pro-inflammatory stimulus. To achieve this, AC16 cells were transfected with a plasmid encoding for *GADD45A* and stimulated with 10 ng/mL of TNF-α for 24 h. As it is displayed in Fig. 2B, TNF-α treatment boosted the expression of pro-inflammatory and pro-fibrotic genes (*CCL2*, collagen type I alpha 1 chain or *COL1A1*, *MMP2*, *MMP9*, *TGFB1*). *GADD45A* overexpression (Supplementary Fig. S1C) did not yield any change in the expression of these genes in the absence of the pro-inflammatory stimulus, but it totally or partially prevented the TNF-α-induced increase in *CCL2*, *COL1A1*, and *MMP2* (Fig. 2B). In contrast, it had no effect on the *MMP9* and *TGFB1* mRNA levels. Overall data point to *GADD45A* overexpression as a factor attenuating the TNF-α-induced pro-inflammatory and pro-fibrotic profile in human cells.

### Deletion of *Gadd45a* gene in knockout mice impacts cardiac metabolism

**Fig. 3:**
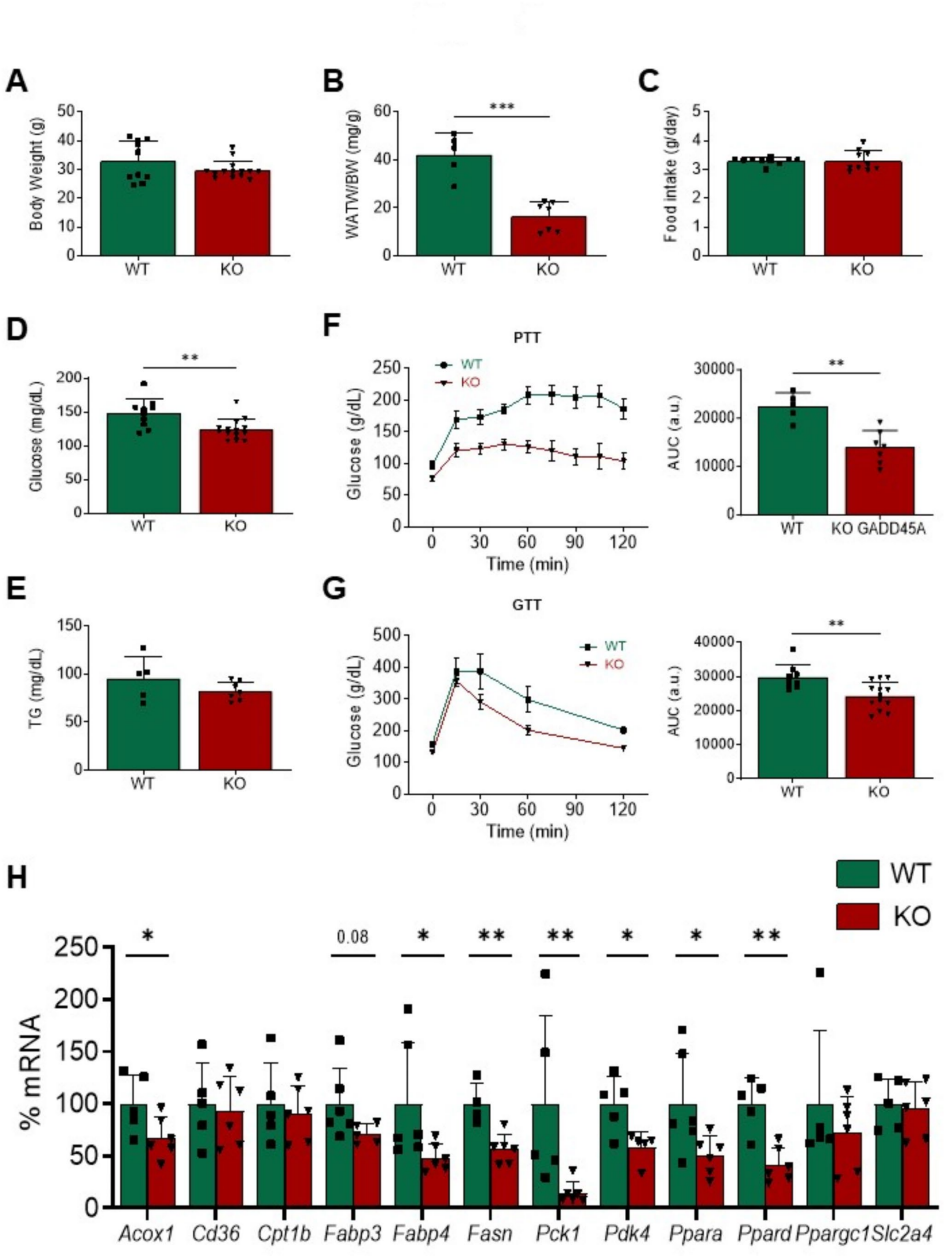
Deletion of *Gadd45a* gene in knockout mice results in hypoglycemia and reduced hepatic gluconeogenesis. Body weight (**A**), epididymal white adipose tissue (WAT) weight to body weight (BW) ratio (**B**), food intake (**C**), and plasma glucose (**D**) and triglyceride (**E**) levels in wild-type (WT) and *Gadd45a* knockout (KO) mice. (**F**) Pyruvate tolerance test and area under the curve (AUC). (**G**) Glucose tolerance test and area under the curve (AUC). (**H**) Relative quantification of the mRNA expression of *Acox1*, *Cd36*, *Cpt1b*, *Fabp3*, *Fabp4*, *Fasn*, *Pck1*, *Pdk4*, *Ppara*, *Ppard*, *Ppargc1*, and *Slc2a4* in the same mice. The mRNA levels were normalized to adenine phosphoribosyl transferase (*Aprt*), and are expressed as a percentage of control samples. Data are presented as the mean ± SD. *p < 0.05, **p < 0.01, *** *p* < 0.001

To fully elucidate the role of GADD45A in the heart, we examined the effects of *Gadd45a* deletion in mice. Considering the above-described role of GADD45A on metabolism in other tissues, we first focused on investigating the putative cardiac metabolic changes produced by suppressing this protein. At sacrifice, *Gadd45a* KO mice displayed a significant reduction in epididymal white adipose tissue mass compared to their WT littermates, despite body weight and food intake were not modified (Fig. 3A-C). Fasting plasma glucose concentration was diminished in KO mice, while plasma triglyceride levels were similar in both strains (Fig. 3D and E). A pyruvate tolerance test suggested that hepatic gluconeogenesis was reduced in mice with *Gadd45a* gene deletion, a fact which might account for the lower plasma glucose levels observed in these animals (Fig. 3F). Despite hypoglycemia, *Gadd45a* suppression in KO mice resulted in improved glucose tolerance compared to WT mice (Fig. 3G).

Molecular analyses revealed that the expression of some genes related to FA uptake and utilization (*Acox1*, acyl-CoA oxidase 1; *Fabp4*, FA binding protein 4; *Fasn*, FA synthase) was significantly reduced in the heart of KO mice (Fig. 3H). The expression of *Fabp3*, the most abundant FA binding protein isoform in the heart, was also slightly reduced, but it did not reach statistical significance. The mRNA levels of PPARα (*Ppara*) and PPARβ/δ (*Ppard*) were also diminished in KO mice and, in agreement with this, the expression of PPAR target genes involved in glucose metabolism, including *Pdk4* and phosphoenolpyruvate carboxykinase 1 (*Pck1*), was also significantly downregulated (Fig. 3H). No changes were observed in the expression of other genes involved in glucose or FA metabolism, including carnitine palmitoyltransferase 1b (*Cpt1b*, cardiac muscle isoform), fatty acid translocase (*Fat*/*Cd36*), or glucose transporter 4 (*Glut4* or *Slc2a4*, solute carrier family 2 member 4) (Fig. 3H).

Cardiac metabolism dysregulation can lead to mitochondrial dysfunction and ensuing overproduction of reactive oxygen species (ROS). Anyhow, despite the observed changes in the expression of metabolism-related genes, no altered mRNA levels of oxidative stress markers (heme oxygenase 1, *Hmox1*; NAD(P)H quinone dehydrogenase 1, *Nqo1*; superoxide dismutase 2, *Sod2*) or mitochondrial function (PPARγ coactivator 1α, *Ppargc1a*) were found in the heart of *Gadd45a* KO mice (Fig. 3H and supplementary Fig. S2A-C).

### Gadd45a deletion induces inflammation and fibrosis in the heart

**Fig. 4:**
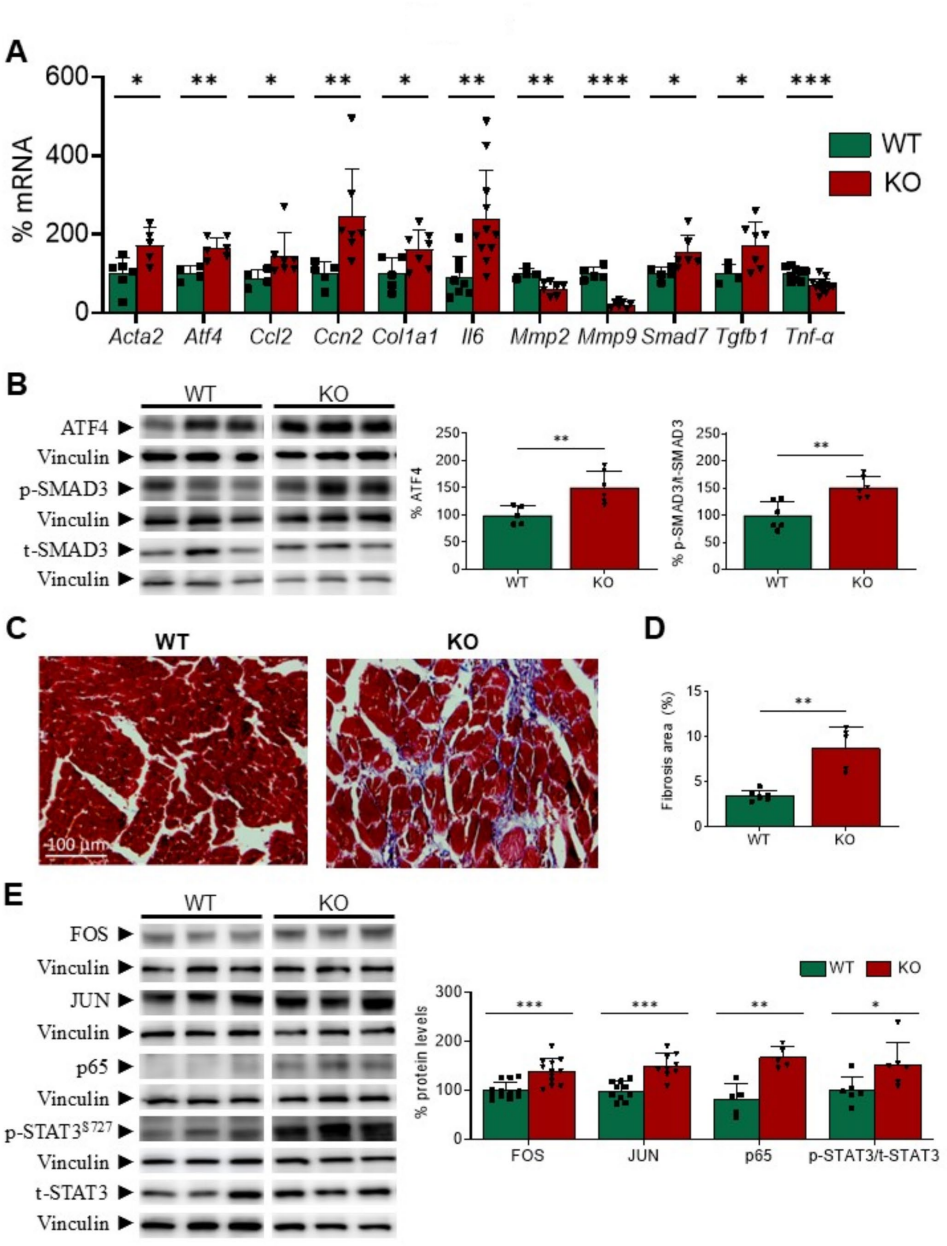
Deletion of *Gadd45a* gene in knockout mice boosts inflammation and fibrosis in the heart. (**A**) Relative quantification of the mRNA expression of *Acta2*, *Atf4*, *Ccl2*, *Ccn2*, *Col1a1*, *Il6*, *Mmp2*, *Mmp9*, *Smad7*, *Tgfb1*, and *Tnf-α* in wild-type (WT) and *Gadd45a* knockout (KO) mice. The mRNA levels were normalized to adenine phosphoribosyl transferase (*Aprt*), and are expressed as a percentage of control samples. (**B**) Western blot analysis showing the protein levels of ATF4 and phospho-SMAD3/total-SMAD3 in the same mice. The graphs represent the quantification of the protein levels normalized to vinculin, and are expressed as a percentage of control samples. Representative images of Mason’s trichrome staining (**C**) and quantification of fibrosis expressed as a percentage (**D**) in the heart of the same animals. (**E**) Western blot analysis showing the protein levels of FOS, JUN, p65, and phospho-STAT3^S727^/total-STAT3 in *Gadd45a* KO and WT mice, as depicted in panel B. Data are presented as the mean ± SD. *p < 0.05, **p < 0.01, *** *p* < 0.001

As it is shown in Fig. 4A, deletion of the *Gadd45a* gene in mice resulted in spontaneous increased expression of some pro-inflammatory and pro-fibrotic markers in the heart with regard to their control littermates, including smooth muscle actin alpha 2 (α*SMA*, or *Acta2*), activating transcription factor 4 (*Atf4*), *Ccl2*, *Ccn2*, *Col1a1*, *Il6*, or *Tgfb1*. This rise was probably not a consequence of increased macrophage infiltration in the heart, as suggested by the absence of changes in the expression of adhesion G protein-coupled receptor E1 (*Adgre1*, or *F4/80*) and *Cd68* antigen (Supplementary Fig. S2D), which are widely used as macrophage-specific markers. In contrast, the mRNA levels of *Tnf-α*, *Mmp2*, and *Mmp9* were reduced (Fig. 4A), whereas those of bone morphogenetic protein 7 (*Bmp7*), collagen type III alpha 1 chain (*Col3a1*), endothelin 1 (*Edn1*), interleukin 1β (*Il1b*), and serine peptidase inhibitor clade E member 1 (*Serpine1*, or plasminogen activator inhibitor 1, PAI-1) were not modified (Supplementary Fig. S2E). In addition to increased *Tgfb1* expression, *Gadd45a* KO mice displayed augmented gene expression (Fig. 4A) and protein levels (Fig. 4B) of the transcription factor ATF4. Further, these mice displayed increased mRNA levels of *Smad7* and increased phosphorylation of SMAD3 (Fig. 4A and B). According to this, Masson’s trichrome staining showed robust cardiac fibrosis, as well as destroyed and disorganized collagen network structure in the interstitial and perivascular areas, in KO mice compared to their WT littermates (Fig. 4C-D).

**Fig. 5:**
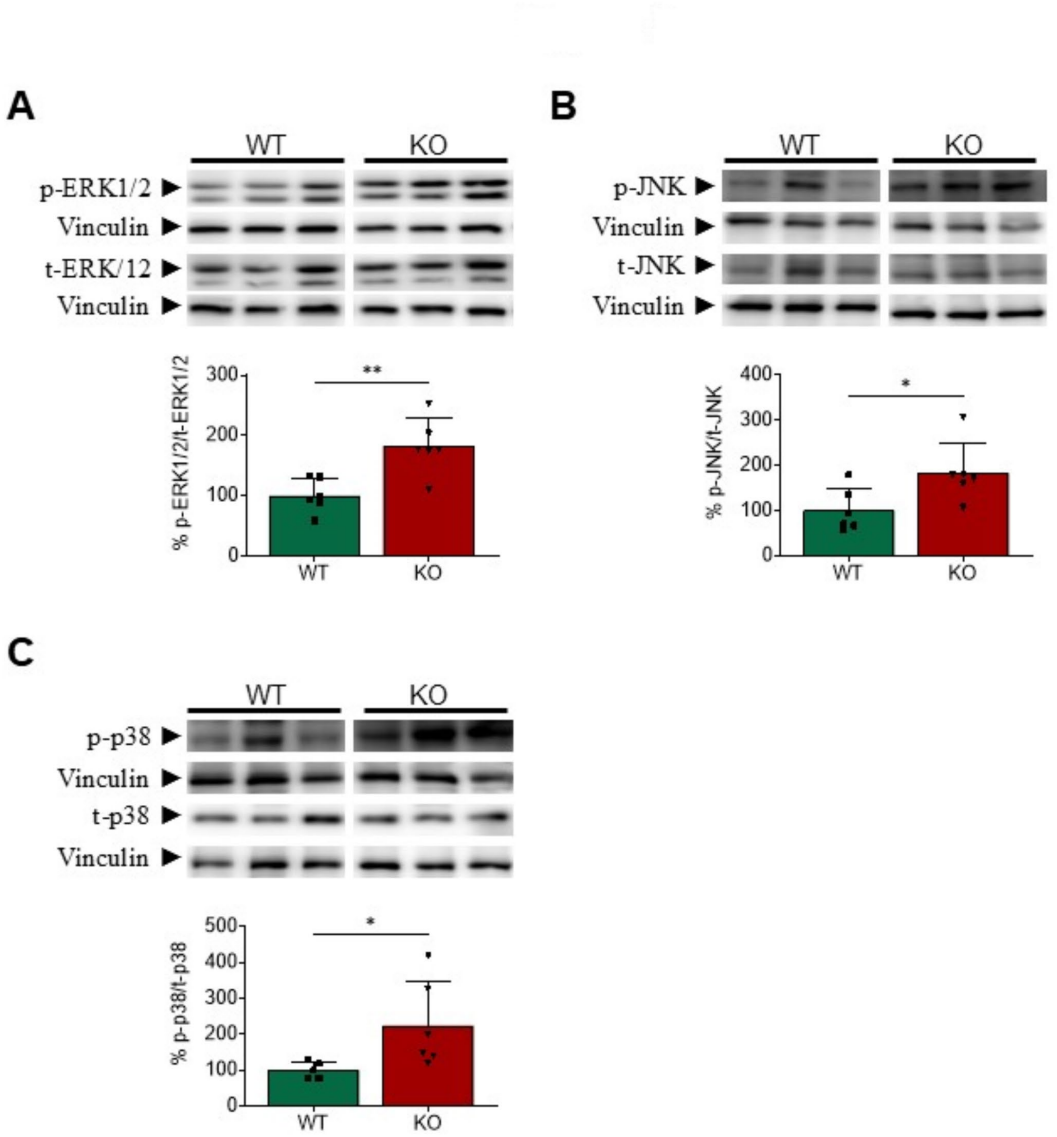
The mitogen-activated protein kinases are induced in the heart of *Gadd45a* knockout mice. Western blot analysis showing the protein levels of phospho-ERK1/2 (extracellular signal-regulated kinase 1/2)/total-ERK1/2 (**A**), phospho-JNK (c-Jun N-terminal kinase)/total-JNK (**B**), and phospho-p38/total-p38 (**C**) mitogen-activated protein kinases (MAPK) in wild-type (WT) and *Gadd45a* knockout (KO) mice. The graphs represent the quantification of the protein levels normalized to vinculin, and are expressed as a percentage of control samples. Data are presented as the mean ± SD. *p < 0.05, **p < 0.01, *** *p* < 0.001

Most pro-inflammatory and pro-fibrotic mediators are transcriptionally regulated by NF-κB and AP-1, which work in cooperation with a complex network of additional transcription factors, including the signal transducer and activator of the transcription 3 (STAT3), to regulate inflammation and fibrosis in the heart. In agreement with this, the hearts of *Gadd45a* KO mice showed increased protein levels of the p65 subunit of NF-κB, and the FOS and JUN subunits of AP-1 (Fig. 4E). STAT3 was also activated in this strain, as demonstrated by the enhanced phosphorylation at Ser727 residue. Next, we sought to determine the potential mechanism by which *Gadd45a* suppression might increase the activity of NF-κB and AP-1. It is widely recognized that several stimuli may induce inflammation, fibrosis, and cardiomyocyte apoptosis through the phosphorylation and subsequent activation of mitogen-activated protein kinases (MAPK) and their downstream targets, NF-κB and AP-1 [37, 38]. Western-blot analyses revealed that the extracellular signal-regulated kinase 1/2 (ERK1/2), c-Jun N-terminal protein kinase (JNK), and p38 MAPK were all phosphorylated, and therefore activated, after suppression of *Gadd45a* in the heart of mice (Fig. 5).

### Gadd45a deletion results in cardiac hypertrophy in mice

**Fig. 6:**
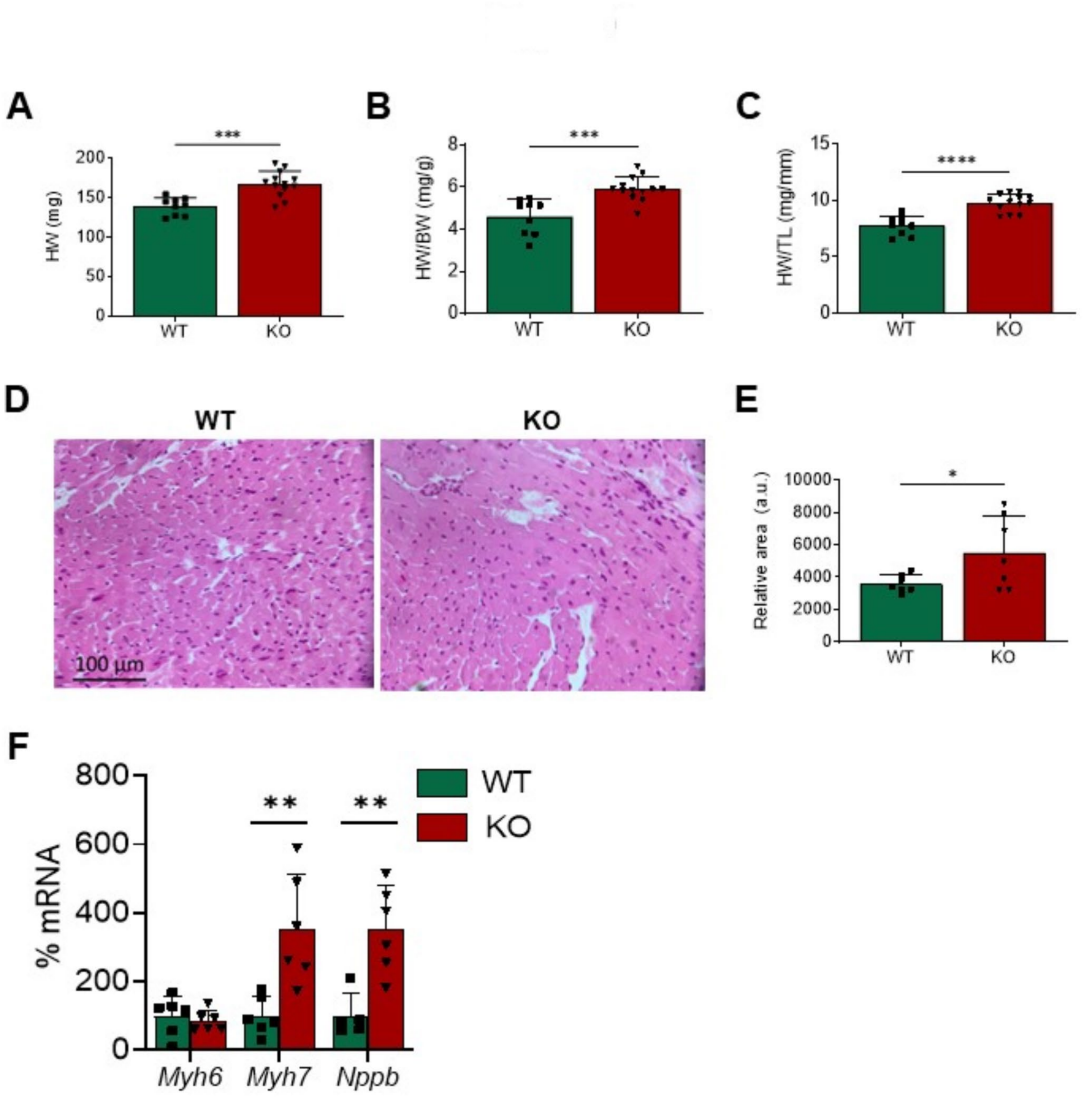
Deletion of *Gadd45a* gene in knockout mice results in substantial cardiac hypertrophy. Heart weight (**A**), and HW to body weight (BW) (**B**) and HW to tibial length (TL) (**C**) ratios in wild-type (WT) and *Gadd45a* knockout (KO) mice. Representative hematoxylin and eosin-stained micrographs (**D**) showing transverse sections from the left ventricle myocardium and quantification of cardiomyocyte cross-sectional areas (**E**) in the same mice. (**F**) Relative quantification of the mRNA expression of *Myh6*, *Myh7*, and *Nppb* in WT and *Gadd45a* KO mice. The mRNA levels were normalized to adenine phosphoribosyl transferase (*Aprt*), and are expressed as a percentage of control samples. Data are presented as the mean ± SD. *p < 0.05, **p < 0.01, *** *p* < 0.001

The heart may adapt to various pathophysiological circumstances by adjusting its relative metabolism of glucose and FA. However, in the event of the loss of this metabolic flexibility, cardiac hypertrophy and ensuing heart failure may arise. In agreement with the metabolic changes depicted above, *Gadd45a* suppression in mice yielded cardiac hypertrophy, as demonstrated by the increase in the heart weight (HW) to body weight (BW; HW/BW) and HW to tibial length (HW/TL) ratios (Fig. 6A-C). This was accompanied by a rise in cardiomyocyte size (Fig. 6D-E). There was also an increase in the expression of cardiac hypertrophy molecular markers, including myosin heavy chain 7 (*Myh7* or β-myosin heavy chain, *β-Mhc*), and natriuretic peptide B (*Nppb* or *Bnp*), in KO mice compared with WT animals, although *Myh6* (*α-Mhc*) was not modified (Fig. 6F). Remarkably, human cardiac AC16 cells with reduced expression of the *GADD45A* gene (transfected with *GADD45A* siRNA) also displayed an increase in *NPPB* expression as compared to control cells (Supplementary Fig. S3A).

**Fig. 7:**
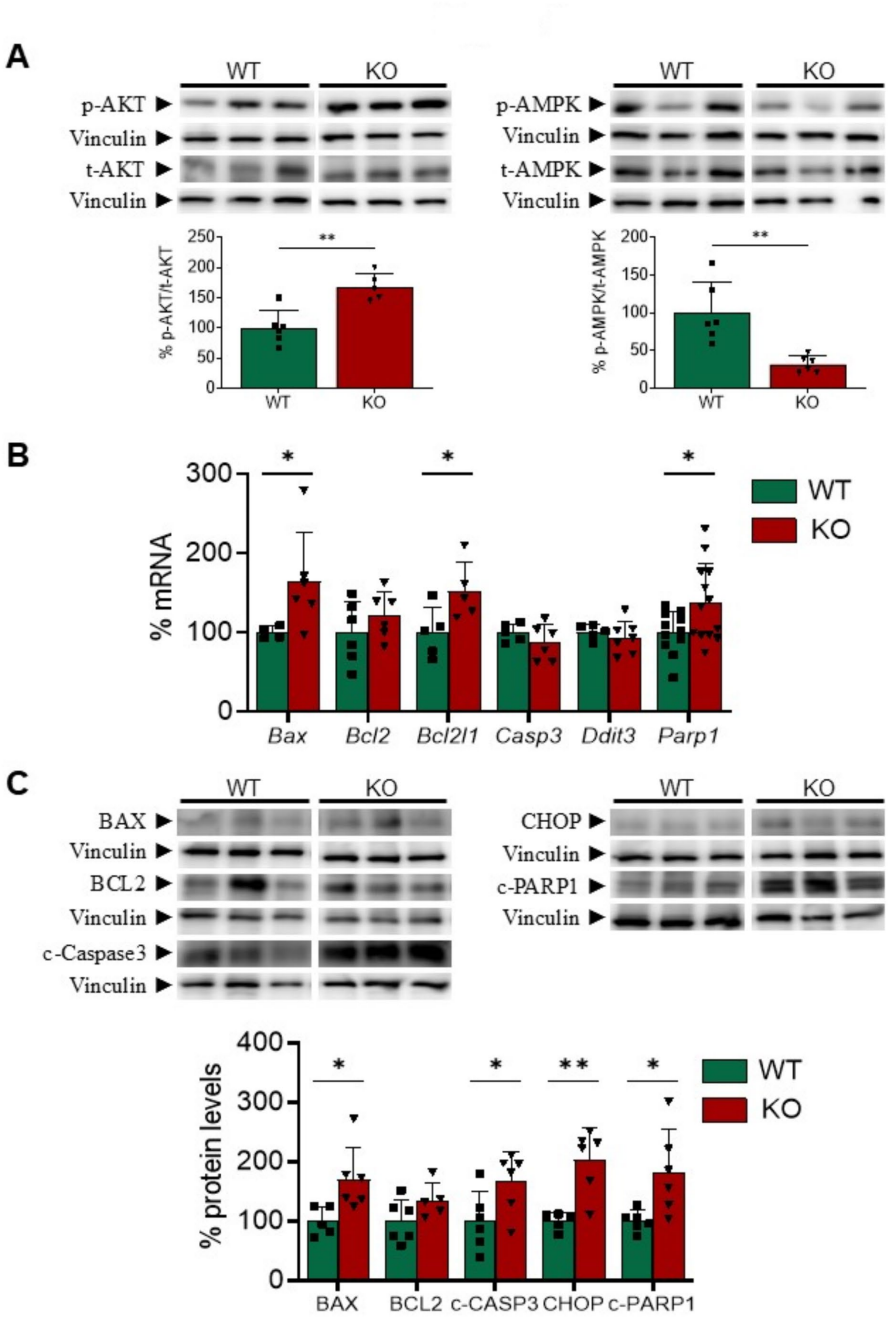
Cardiac apoptotic markers are induced in the heart of *Gadd45a* knockout mice. (**A**) Western blot analysis showing the protein levels of phospho-AKT/total-AKT, and phospho-AMPK/total-AMPK in wild-type (WT) and *Gadd45a* knockout (KO) mice. The graphs represent the quantification of the protein levels normalized to vinculin, and are expressed as a percentage of control samples. (**B**) Relative quantification of the mRNA expression of *Bax*, *Bcl2*, *Bcl2l1*, caspase 3 (*Casp3*), *Ddit3*, and *Parp1* in WT and *Gadd45a* KO mice. The mRNA levels were normalized to adenine phosphoribosyl transferase (*Aprt*), and are expressed as a percentage of control samples. **(C**) Western blot analysis showing the protein levels of BAX, BCL2, cleaved caspase 3, CHOP and cleaved PARP1 in the same mice, as depicted in panel A. Data are presented as the mean ± SD. *p < 0.05, **p < 0.01, *** *p* < 0.001

We next explored the potential mechanism involved in the cardiac hypertrophy arising as a consequence of *Gadd45a* deletion. During ER stress, the protein kinase R-like ER kinase (PERK)/ATF4 branch of the unfolded protein response (UPR) may prevent cardiomyocyte hypertrophy by reducing the accumulation of misfolded proteins [39]. However, we found that ATF4 expression and protein levels were increased in KO mice (Fig. 4A and B). Other markers of the different branches of the UPR elicited during ER stress, including *Atf3* and *Hspa5* (heat shock protein family A, Hsp70, member 5, commonly referred to as BiP/GRP78, binding immunoglobulin protein/78 kDa glucose-regulated protein) were not increased in KO mice either (Supplementary Fig. S3B and C). Therefore, it is unlikely that ATF4, or even ER stress, are contributing to the development of cardiac hypertrophy in our model. Other effector pathways regulating cardiomyocyte size are also involved in the transcriptional regulation of *GADD45A*, including those controlled by NFAT, the protein kinase B (PKB or AKT), and MAPK [7, 39]. All these pathways regulate protein synthesis and degradation too. As reported above (Fig. 5), *Gadd45a* KO mice had increased activity of the MAPK signaling pathways in the heart, but they also displayed activation of AKT, as demonstrated by the increased phosphorylation of this kinase (Fig. 7A). In agreement with the augmented AKT activity, phosphorylation of the Thr172 residue of the AMP-activated protein kinase (AMPK) was reduced (Fig. 7A).

Besides cell enlargement, another important contributor to cardiac hypertrophy is the balance between cardiomyocyte survival and cell death. Since GADD45A is regarded an important modulator of apoptosis, and we had observed an activation of the pro-apoptotic p38 and JNK pathways, we also explored the occurrence of apoptosis in our model. The mRNA levels of *Bax* (BCL2 associated X), *Bcl2l1* (B-cell CLL/lymphoma 2, BCL2, like 1 or *Bcl-XL*), and *Parp1* (poly ADP ribose polymerase 1) were increased in the heart of *Gadd45a* KO mice, while those of *Bcl2*, caspase 3 (*Casp3*), and DNA damage inducible transcript 3 (*Ddit3*, which encodes for the C/EBP homologous protein, CHOP) did not change (Fig. 7B). Western-blot analysis revealed that the protein levels of BAX, cleaved caspase 3, CHOP, and cleaved PARP1 were all increased in KO mice (Fig. 7C), whereas those of BCL2 were not, overall suggesting that cardiomyocyte apoptosis was upregulated in the heart of these mice and, therefore, it could hardly contribute to cardiac hypertrophy.

### Gadd45a deletion impacts cardiac function in mice

We finally aimed to examine, by means of transthoracic echocardiography, whether dysregulation yielded by *Gadd45a* suppression resulted in changes in cardiac morphology and performance in KO mice. Heart rate was comparable between KO and WT animals but, since body temperature was not continuously monitored during echocardiographic studies, it cannot be ruled out that the lack of changes in heart rate was due to body temperature differences between experimental groups, since GADD45A plays an important role in energy metabolism [40, 41] and non-shivering thermogenesis [18]. Reduced expression of *Gadd45a* induced hypertrophy of the left ventricle, with an increase in wall thicknesses (IVSd, interventricular septal wall thickness at end-diastole; PWTd, left ventricular posterior wall thickness at end-diastole) and, consequently, the mass of the chamber (estimated LV mass or eLV mass; Table 1). The geometry of the chamber, reflected in the diastolic relative thickness of the posterior wall (rLV PWTd), suggests a pattern of concentric hypertrophy. LV systolic function was preserved in KO animals, as judged by the maintained EF values (Table 1). There were no differences in the deceleration time of the E wave, suggesting no differences in diastolic function. Cardiac output was also increased in *Gadd45a* KO mice, probably due to a slightly higher heart rate, and predictably larger left ventricular outflow tract as corresponds to a larger chamber. The higher peak flow velocity (Ao PV) in the KO group may be due to the reduction in ejection time, caused by a slightly higher heart rate.

**Table 1:** Echocardiographic data of wild-type (WT) or *Gadd45a* knockout (KO) male mice

Parameter	Group	
	WT	Gadd45a KO
EF (%)	50.5 ± 12.5	43.7 ± 7.2
FS (%)	30.2 ± 9.6	27.5 ± 4.9
IVSd (mm)	0.71 ± 0.12	0.89 ± 0.17**
IVSs (mm)	0.95 ± 0.15	1.09 ± 0.13*
LV PWTd (mm)	0.73 ± 0.15	0.88 ± 0.16*
LV PWTs (mm)	0.89 ± 0.11	1.09 ± 0.27*
eLV mass (mg)	77.7 ± 20.0	101.4 ± 23.4*
rLV PWTd (ratio)	0.39 ± 0.12	0.45 ± 0.11*
LV EDD (mm)	3.82 ± 0.68	3.89 ± 0.25
LV ESD (mm)	2.73 ± 0.67	2.82 ± 0.30
CO (mL/min)	5.72 ± 1.20	7.31 ± 1.00*
*E*-DT (msec)	24.6 ± 3.8	27.0 ± 1.5
Asc. Ao diameter (mm)	1.37 ± 0.04	1.39 ± 0.10
Ao VTI (mm2)	4.49 ± 0.94	5.26 ± 0.72*
Ao PV (mm/sec)	0.89 ± 0.23	1.21 ± 0.25**
Heart rate (bpm)	361 ± 72	409 ± 54

Data indicate mean ± SD (*n* = 11–13 mice per group)

Abbreviations: Ao VTI, aortic velocity time integral; Ao PV, aorta peak velocity; Asc. Ao. diameter, ascending aorta diameter; CO, cardiac output; *E*-DT, deceleration time of early-diastolic transmitral flow velocity wave; EF, ejection fraction; eLV mass, estimated left ventricular mass; FS, fractional shortening; IVSd and IVSs, interventricular septal wall thickness at end-diastole and end-systole; KO, knockout; LV, left ventricle; LV EDD, left ventricular end-diastolic diameter; LV ESD, left ventricular end-systolic diameter; LV PWTd and PWTs, left ventricular posterior wall thickness at end-diastole and end-systole; rLV PWTd, relative left ventricular posterior wall thickness at end-diastole; WT, wild-type. *P < 0.05, **P < 0.01 and ***P < 0.001 vs. WT

## Discussion

GADD45A is a multifaceted protein associated with stress signaling and cellular injury response by means of its ability to control cell migration, DNA repair, and cell apoptosis, survival and senescence [7]. Furthermore, recent studies point to additional roles for GADD45A, including the regulation of catabolic and anabolic pathways, or the prevention of inflammation, fibrosis, apoptosis, and oxidative and ER stress in several tissues [16, 42]. Data presented in this study highlight an important function for GADD45A in the heart, since it would play a protective role by preventing inflammation, fibrosis and apoptosis, thus preserving cardiac function and performance. However, how GADD45A regulates these processes still needs to be fully clarified in future studies. Another limitation of the study is that we have not investigated the effects of GADD45A deletion in female mice, nor the occurrence of gender-differences in the development of cardiac hypertrophy. Indeed, although current research indicates that GADD45A does not present significant sex-related differences in its expression and function [43, 44], several studies suggest that women and female mice show less inflammation, fibrosis, and apoptosis upon heart failure and cardiac hypertrophy [45].

Our data demonstrate that *Gadd45a* suppression triggered systemic metabolic changes, including plasma hypoglycemia and diminished hepatic gluconeogenesis, which may undoubtedly affect the heart. These combined effects on metabolism may compromise the ability of the heart to generate sufficient energy, potentially leading to decreased cardiac efficiency and function, and eventually contributing to the progression of heart failure. Cardiac metabolism is mostly regulated by the PPAR transcription factor family, which includes three isoforms, PPARα, PPARβ/δ and PPARγ [46]. PPARα and PPARβ/δ are the predominant isoforms in the heart, where they share some overlapping functions in regulating lipid metabolism and glucose homeostasis [47]. Interestingly, we observed reduced mRNA levels of *Ppara* and *Ppard*, and their target genes *Pdk4* and *Pck1*, in *Gadd45a* KO mice. PCK1 participates in myocardial glucose homeostasis by modulating both glycolysis and gluconeogenesis [48], and its dysregulation contributes to the development of cardiac hypertrophy [49]. Thus, reduced *Pck1* expression in the heart might result in impaired gluconeogenesis, limiting the availability of glucose and potentially leading to a deficit in energy supply, especially under hypoglycemia conditions. Regarding PDK4, it is responsible for the phosphorylation-mediated inactivation of the pyruvate-dehydrogenase complex (PDC), which catalyzes the rate-limiting step of glucose oxidation, thus decreasing glucose oxidation while allowing increased fatty acid β-oxidation [46, 50]. Consequently, the reduced *Pdk4* expression in the heart of KO mice might be aimed at activating the PDC to enhance glucose oxidization and, thus, counteract the reduced glucose supply [51]. This is not the first study reporting a role for GADD45A on regulating metabolism, since it is known that its knockdown in adipose tissue improves insulin sensitivity, glucose uptake, and energy expenditure, and exerts a PPARγ-dependent adipogenic effect [18]. In the liver, GADD45A triggers AMPK activity to induce the expression of genes related to glucose uptake, glycolysis, glycogenolysis, FA β-oxidation, and lipolysis, while inhibiting the gluconeogenesis and the biosynthesis of FA [52]. Another study demonstrated that GADD45A and PPARα interact in the heart of diabetic rats [28], although the functional consequences of this interaction were not elucidated. The same study reported that the diabetic heart exhibits reduced GADD45A protein levels, and this might account for to the metabolic disturbances that characterize this condition, which are accompanied by local inflammation, fibrosis, ER stress, and cardiomyocyte apoptosis. Interestingly, PPARα activity is also diminished in the diabetic heart and its downregulation is also characteristic of cardiac pathological hypertrophy and failure in rodent models [53]. This effect over PPARs is not surprising, since GADD45 proteins contain the LXXLL signature, a motif which is known to be involved in protein‐protein interactions, and GADD45 proteins are known to interact and activate the three PPAR subtypes [54, 55].

Adult cardiac myocytes mostly rely on free FA as the preferred energy substrate. However, they must use alternative fuel sources, including glucose, lactate, or ketone bodies, to adapt to diverse pathophysiological circumstances, and to warrant an alternative and constant energy source [56]. Hence, in the context of hypoglycemia, an upregulation of genes related to cardiac FA uptake and utilization would be expected to occur. On the contrary, we observed a reduction in the expression of genes related to cardiac lipid catabolism in *Gadd45a* KO mice, as well as in the expression of *Ppara* and *Ppard*, which encode for the nuclear receptors responsible for the transcriptional regulation of these genes. We do not know the reasons for this discrepancy, but a previous study has already reported that severe hypoglycemia may result in lipid metabolism inhibition and aggravated cardiac injury [57]. It cannot be disregarded either that downregulation of PPAR activity in the heart was aimed to increase glucose utilization, but this might decrease ATP synthesis efficiency and contribute to cardiac remodeling [58]. In fact, *Pdk4* was also reduced in *Gadd45a* KO mice, and reduced PDK4 activity in cardiac cells is often accompanied by depressed cardiac performance, cardiac hypertrophy and heart failure [59].

Myocardial injury caused by inflammation is actively involved in the development of interstitial fibrosis, which is a hallmark of myocardial remodeling and is considered a primary determinant of worsened cardiac performance [60]. *Gadd45a* KO mice showed an increase in the gene expression and protein accumulation of several pro-inflammatory and pro-fibrotic markers in the heart. Cytokines and chemokines exert several deleterious autocrine effects via downstream activation of AP-1 and NF-κB [50, 61]. AP-1 is a heterodimeric transcription factor mainly composed of members belonging to the JUN, FOS, and ATF partner families that, through the stimulation of its target genes (*Ccn2*, *Col1a1*), deregulates the extracellular matrix composition and structure, eventually leading to cardiac fibrosis and decreased contractility [62]. These transcription factors, together with STAT3, cause changes in the extracellular matrix and decrease contractility, inducing cardiomyocyte hypertrophy and fibrosis. Thus, it is not surprising that the hearts of *Gadd45a* KO mice displayed increased protein levels of the p65 subunit of NF-κB, the FOS and JUN subunits of AP-1, as well as activation of STAT3. This latter result fits well with a previous study demonstrating that GADD45A suppresses the transcriptional activity of STAT3, by this means blocking angiogenesis and tumorigenesis [63]. In the heart, STAT3 participates in processes related to inflammation, fibrosis and cell death, and, in fact, it may also boost NF-κB activity [31]. The mechanism responsible for the upregulation of AP-1 and NF-κB in our KO mice probably involved the activation of ERK1/2, JNK and p38 MAPK, since we found that all three MAPK were phosphorylated, and thus activated, in the heart of these mice. Interestingly, all three MAPK may positively regulate *Gadd45a* transcription [12], and are also involved in NF-κB and AP-1 activation [37, 38]. Our data contrast with previous studies demonstrating that GADD45A activates the p38 and JNK pathways [63, 64], but it is also true that GADD45A may also act as a negative regulator of ERK1/2 and p38 MAPK activity in some cell types or under particular circumstances [15, 65]. In particular, deletion of *Gadd45a* in mice boosted neuroinflammation, thus contributing to the neurodegenerative process, and this was characterized by an hyperactivation of ERK1/2 and NF-κB signaling pathways [65]. Anyhow, we cannot disregard that the activation of ERK1/2, JNK and p38 MAPKs observed in our study was related to the increase in pro-inflammatory and pro-fibrotic cytokines, and subsequent activation of NF-κB and AP-1, or to compensatory mechanisms aimed to upregulate *Gadd45a* expression in the heart of KO mice.

It is worth noting the activation of the TGFβ1 signaling pathway observed in the heart of KO mice. TGFβ1 is a multifunctional cytokine induced after myocardial injury that is involved in the regulation of inflammation, extracellular matrix deposition, and cell fate. In cardiomyocytes, it may be transcriptionally induced by ATF4, overall driving progressive cardiac fibrosis in diseases such as arrhythmogenic [66] and diabetic [67] cardiomyopathies. Binding of TGFβ1 to its receptor triggers the phosphorylation of the downstream effector proteins SMAD2/3 (mothers against decapentaplegic homolog 2 and 3), which, after forming heterodimeric complexes with SMAD4, enter the cell nucleus to transcriptionally promote cardiac fibrosis development [68, 69]. The inhibitory SMAD7, which is also transcriptionally induced upon binding of TGFβ1 to its receptor, negatively regulate the TGFβ cascade by competing with SMAD2/3 for their binding to the TGFβ receptor and SMAD4 [69]. As stated above, ATF4 also enhances *Gadd45a* transcription under ER stress or other stressors, by directly interacting with an ATF/CREB (cAMP response element binding protein)-related binding element within its promoter [7]. The hearts of *Gadd45a* KO mice displayed, besides increased *Tgfb1* expression, augmented expression and protein levels of ATF4, increased mRNA levels of *Smad7*, and increased phosphorylation of SMAD3, thus overall reinforcing the occurrence of a pro-fibrotic state in these mice, which was confirmed by the Masson’s trichrome staining. In a similar way, the downregulation of GADD45A, together with the activation of the TGFβ signaling pathway, has been observed in fibrotic livers of murine models, while GADD45A overexpression downregulates the expression of extracellular matrix proteins and may counteract hepatic fibrosis by inhibiting the TGF‐β/SMAD signaling pathway [70].

In our study, *Gadd45a* suppression in KO mice resulted in robust cardiac hypertrophy. This was not surprising, since GADD45A has been shown to induce muscle atrophy [20], and a recent in silico study had already pointed to GADD45A as a hub protein underlying hypertrophic cardiomyopathy in humans [27]. Skeletal muscle atrophy is a debilitating condition caused by diverse stresses, including aging, diabetes, starvation, or muscle denervation, which is often characterized by a reduction in AKT signaling and an increase in ATF4 and caspase 3 activity, and autophagy [7, 20, 71, 72]. These same stressors are also capable of inducing *GADD45A* expression, which will cause muscle atrophy in a process at least partially regulated by ATF4 [7, 20]. To carry out its pro-atrophying effect, GADD45A transcriptionally represses genes involved in anabolic signaling, protein synthesis, glycolysis, oxygen delivery, mitochondrial biogenesis, citric acid cycle, and oxidative phosphorylation, but induces genes involved in autophagy-mediated proteolysis [7]. In addition, GADD45A cooperates with other transcriptional regulators, such as forkhead box protein O (FOXO) and NF‐κB, to bring about these effects [73]. It has been already established that *Gadd45a* KO mice have greater ER stress in the liver compared with WT mice [16]. In the event of ER stress, the UPR pathway aims to counteract cell stress by regulating the protein turnover but, if the stress is not resolved, protein accumulation may arise. It is worth mentioning that cardiomyocyte hypertrophy often occurs when protein synthesis overcomes its degradation [7]. Further, ATF4 may prevent cardiomyocyte hypertrophy [39] and, as stated above, ATF4 is also relevant in controlling *Gadd45a* transcription. However, our results rule out a role for ATF4, or even ER stress, in the development of cardiac hypertrophy in our model.

Adult cardiomyocytes are refractory to proliferation and, thus, any change in heart size often arises as a consequence of hypertrophy or atrophy of the individual cells. The heart of *Gadd45a* KO mice displayed an increase in the activity of AKT and MAPK signaling pathways, which, besides being involved in the transcriptional regulation of *GADD45A*, also regulate cardiomyocyte size [7]. Under hypoglycemia conditions, as observed in *Gadd45a* KO mice, insulin secretion by pancreatic β cells is increased. The binding of this hormone to its receptor in cardiomyocytes activates the phosphatidylinositol 3-kinase (PI3K)/AKT pathway to promote glucose utilization [50]. However, and in contrast to skeletal muscle, liver, or adipose tissue, insulin signaling in the heart inhibits the activity of AMP-activated protein kinase (AMPK) [74], a master regulator of metabolism that also promotes glucose uptake and utilization. This inhibition is dependent on the AKT-mediated phosphorylation of AMPK Ser485 or Ser491 residues, which hinders the liver kinase B1 (LKB1, also known as serine/threonine kinase 11 or STK11) activity on the major regulatory site of AMPK, the Thr172 residue of its α catalytic subunit [75]. Consequently, and in agreement with the augmented AKT activity, phosphorylation of the Thr172 residue of AMPK was reduced in the heart of *Gadd45a* KO mice. This reduction is harmful, since AMPK activity is regarded as cardioprotective, because it is a master regulator of metabolism that protects the heart against ischemic injury, oxidative stress-induced cell death and cardiac hypertrophy [76]. Remarkably, children with hypoglycemia due to congenital hyperinsulinism displayed cardiac structural lesions with persistent ventricular hypertrophy [77]. Likewise, resveratrol exerts its anti-hypertrophic effect in the heart by activating AMPK and inhibiting AKT [78], while AMPK inactivation correlates with cardiac hypertrophy and remodeling in rodent models with obesity induced by high-fat or high-fructose diets [79, 80]. To sum up, increased activity of AKT and MAPK, together with an inhibition of the AMPK signaling pathway, might contribute to the cardiac hypertrophy phenotype observed in *Gadd45a* KO mice.

Finally, apoptosis might be another important contributor to the cardiac hypertrophy observed in *Gadd45a* KO mice, since excessive apoptosis can lead to a loss of cardiomyocytes, which the heart may try to compensate for by enlarging the remaining cells and, thus, cause cardiac hypertrophy [81]. Aside its well-established pro-apoptotic and tumor suppressor activity, a pro-survival role has also been reported for GADD45A in some studies [5, 82–84]. In accordance with this, the lack of *Gadd45a* has been related to both reduced or increased apoptosis in different cell types [6, 8, 9]. The anti-apoptotic effect of GADD45A is related to its ability to inhibit the activity of β-catenin, and subsequent *MMP10* expression [85], and to the inhibition of the mitochondrial apoptotic pathway [5]. On the other hand, and similar to what occurs in the diabetic heart, excess FA utilization owing to the shortage in glucose supply might result in the accumulation of toxic lipid intermediates, which may favor myocyte apoptosis and myocardial fibrosis [46, 47, 58]. In our hands, the heart of mice with deleted *Gadd45a* expression displayed an activation of the p38 and JNK pathways and an elevation of several pro-apoptotic markers. This is not surprising, since the rate of cardiomyocyte apoptosis can significantly upsurge during hypertrophic heart disease, and is an important mechanism for cardiac remodeling [86]. The downregulation of FABP3 might also contribute to the increased apoptosis in our model, since this FA transport protein is a PPAR target that also exerts an anti-apoptotic effect in cardiac cells [87]. Analogous to our results, muscle atrophy due to denervation provoked the upregulation of GADD45A and subsequent reduction in the apoptosis of myofibers by blocking the NF-κB signaling pathway [73]. Thus, it is feasible that *Gadd45a* suppression de-represses apoptosis through NF-κB activation and subsequent inflammation. It cannot be disregarded either that decreased oxygen delivery to the cardiomyocyte because of defective capillary angiogenesis [86] may contribute to the rise in apoptosis observed in KO mice.

## Conclusion

The present study demonstrates that reduced GADD45A activity in mice results in cardiac inflammation, fibrosis, and hypertrophy, which, together with metabolic dysregulation, negatively impact cardiac morphology and function. Overall data identify GADD45A as an essential factor during heart disease. Further, overall picture in humans fits well with the mouse findings. Hence, GADD45A positively correlates with functional variables or biochemical effectors indicative of benign remodeling and inversely with clinical, echocardiographic or biochemical variables that are associated with a greater and less reversible remodeling. Also, the impact that GADD45A has on glucose uptake and insulin sensitivity point to that targeting GADD45A might be therapeutically useful to counteract some of the effects contributing to obesity and diabetes. Further studies are warranted to examine the role of GADD45A in stressed conditions, such as in mice subjected to transverse aortic constriction, which will provide valuable information regarding the function of this protein in cardiac hypertrophy.

## Electronic supplementary material

Below is the link to the electronic supplementary material.

Supplementary Material 1

Supplementary Material 2

## Data Availability

The source data for this study are available as a Source Data file or from the corresponding author upon reasonable request.

## Abbreviations

ACOX1: acyl-Co A oxidase 1
ADGRE1: adhesion G protein-coupled receptor E1 (F4/80)
AKT: protein kinase B (PKB)
AMPK: AMP-activated protein kinase
Ao PV: aorta peak velocity
Ao VTI: aortic velocity time integral
AP-1: activator protein-1
APRT: adenine phosphoribosyl transferase
ATF: activating transcription factor
BAX: BCL2 associated X
BCL2: B-cell CLL/lymphoma 2
BCL-XL: BCL2 like 1 (BCL2L1)
BNP (NPPB): natriuretic peptide B
BW: body weight
CCL2: chemokine (C-C motif) ligand 2 (MCP1, monocyte chemoattractant protein)
CCN2: cellular communication network factor 2 (CTGF, connective tissue growth factor)
CHOP: C/EBP homologous protein
COL1A1: collagen type I alpha 1 chain
COL3A1: collagen type III alpha 1 chain
CPT1b: carnitine palmitoyltransferase 1b
DDIT3: DNA damage inducible transcript 3
EDN1: endothelin 1
EF: ejection fraction
EGR1: early growth response protein 1
eLV mass: estimated left ventricular mass
ER: endoplasmic reticulum
ERK: extracellular signal-regulated kinase
FA: fatty acid
FABP: fatty acid binding protein
FASN: fatty acid synthase
FAT: fatty acid translocase (CD36)
FS: fractional shortening
GADD45: growth arrest and DNA damage inducible 45
HMOX1: heme oxygenase 1
HW: heart weight
IL: interleukin
IVSd or IVSs: interventricular septal wall thickness at end-diastole or end-systole
JNK: c-Jun N-terminal kinase
KO: knockout
LV: left ventricle
LV EDD or ESD: left ventricular end-diastolic or end-systolic diameter
MAPK: mitogen-activated protein kinase
MAPSE: mitral annular plane systolic excursion
α-MHC: α-myosin heavy chain (MYH6, myosin heavy chain 6)
β-MHC: β-myosin heavy chain (MYH7)
MMP: matrix metalloproteinase or metallopeptidase
NF-κB: nuclear factor-κB
NFAT: nuclear factor of activated T-cells
NFYA: nuclear transcription factor Y subunitα
NQO1: NAD(P)H quinone dehydrogenase 1
PARP1: poly ADP ribose polymerase 1
PCK1: phosphoenolpyruvate carboxykinase 1 (PEPCK)
PDC: pyruvate-dehydrogenase complex
PDK4: pyruvate dehydrogenase kinase 4
PERK: protein kinase R-like ER kinase
PI3K: phosphatidylinositol 3-kinase
POU2F1: POU domain class 2 transcription factor 1 (OCT-1, octamer-binding protein 1)
PPAR: peroxisome proliferator-activated receptor
PPARGC1a: PPARγ coactivator 1α (PGC-1α)
PWTd or PWTs: left ventricular posterior wall thickness at end-diastole or end-systole
ROS: reactive oxygen species
siRNA: small interfering RNA
SLC2a4: solute carrier family 2 member 4 (GLUT4, glucose transporter 4)
αSMA: smooth muscle actin alpha 2 (ACTA2)
SMAD: mothers against decapentaplegic homolog
SOD2: superoxide dismutase 2
STAT3: signal transducer and activator of transcription 3
TGFB1/TGFβ: transforming growth factor β1
TL: tibial length
TNF-α: tumor necrosis factor-α
UPR: unfolded protein response
WT: wild-type
WT1: Wilms tumor protein 1
