# Supplementary material for: GADD45A suppression contributes to cardiac remodeling by promoting inflammation, fibrosis and hypertrophy
Source: Cell Mol Life Sci. 2025 Apr 30;82(1):189. doi: 10.1007/s00018-025-05704-x (PMC12040809; doi:10.1007/s00018-025-05704-x)
Supplement: Supplementary file 1 — Supplementary Material 1 [file 18_2025_5704_MOESM1_ESM.pdf]

SUPPLEMENTARY FILE 1 to:

GADD45A contributes to cardiac remodeling by promoting inflammation, fibrosis and hypertrophy

Adel Rostami, Xavier Palomer, Javier Pizarro-Delgado, Lucía Peña, Mònica Zamora, Marta Montori-Grau, Emma Barroso, Brenda Valenzuela-Alcaraz, Fàtima Crispi, Jesús M. Salvador, Raquel García, María A. Hurlé, Francisco Nistal, Manuel Vázquez-Carrera

Correspondence to: Xavier Palomer (xpalomer@ub.edu; ORCID: 0000-0001-7647-9984) and Manuel Vázquez-Carrera (mvazquezcarrera@ub.edu; ORCID: 0000-0001-7138-8207), Department of Pharmacology, Toxicology and Therapeutic Chemistry, Faculty of Pharmacy and Food Sciences, University of Barcelona, Av. Joan XXIII 27-31, E-08028, Barcelona, Spain. Tel: +34 934024531.

Supplementary Table 1 List of primers used for quantitative real-time RT-PCR.

Gene	Forward (5'-3')	Reverse (5'-3')
	**Mouse**	
Acox1	TCTGGAGATCACGGGCACTT	TTTCCAAGCCTCGAAGATGAG
Acta2	TGCTGAGACAGAGGCACCACTGAA	CAGTTGTACGTCCAGAGGCATAG
Adgre1	AAGGCCCAGGAGTGGAATGT	GGATATTGGTGCAGACTGAGTTAGG
Atf4	AGCAAAACAAGACAGCAGCC	ACTCTCTTCTTCCCCCTTGC
Aprt	CAGCGGCAAGATCGACTACA	AGCTAGGGAAGGGCCAAACA
Bax	CAAGAAGCTGAGCGAGTGTC	GCAATCATCCTCTGCAGCTC
Bcl2	GTGGATGACTGAGTACCT	CCAGGAGAAATCAAACAGAG
Bcl2l1	AATGACCCACCTAGAGCCTTGG	GCTGCATTGTTCCCGTAGAGA
Bmp7	ATCGTCCAGACACTGGTTCA	AGCTGTCGTCGAAGTAGAGG
Caspase3	AAGGAGCAGCTTTGTGTGTG	GGCAGGCCTGAATGATGAAG
Ccl2	GCTGGAGAGCTACAAGAGGATCA	CTCTCTCTTGAGCTTGGTGACAAA
Ccn2	GCAGCGGTGAGTCCTTCC	AATGTGTCTTCCAGTCGGTAGG
Cd36	AAGCCAGCTAGAAAAATAGAAGCATT	AGTCTCATTTAGCCACAGTATAGGTACAA
Cd68	GGACTACATGGCGGTGGAATA	GATGAATTCTGCGCCATGAA
Col1a1	GACTGGAAGAGCGGAGAGTACTG	CCTTGATGGCGTCCAGGTT
Col3a1	GCCCACAGCCTTCTACAC	CCAGGGTCACCATTTCTC
Cpt1b	GGCTGCCGTGGGACATT	TGCCTTGGCTACTTGGTACGA
Ddit3	CGAAGAGGAAGAATCAAAAACCTT	GCCCTGGCTCCTCTGTCA
Edn1	ACTTCCCAATAAGGCCACAG	TACTTTGGGCCCTGAGTTCT
Fap3	CATGAAGTCACTCGGTGTGG	CTGTCACCTCGTCGAACTCT
Fabp4	AAGAAGTGGGAGTGGGCTTT	ATGATCATGTTGGGCTTGGC
Fasn	GCTGCGGAAACTTCAGGAAAT	AGAGACGTGTCACTCCTGGACTT
Gadd45a	AATATGACTTTGGAGGAATTC	ATTCGGATGCCATCACCGTTC
Gapdh	TGTGTCCGTCGTGGATCTGA	CCTGCTTCACCACCTTCTTGA
Hmox1	GAAAATGTGATTCACTCCTCTGACA	CCTTGGTGGCCTCCTTCAA
Hspa5	CAGATCTTCTCCACGGCTTC	GCAGGAGGAATTCCAGTCAG
Il1b	AATCTATACCTGTCCTGTGTAATGAAAGAC	TGGGTATTGCTTGGGATCCA
Il16	ACACATGTTCTCTGGGAAATCGT	AAGTGCATCATCGTTGTTCAT
Mmp2	ACCCAGATGTGGCCAACTAC	TACTTTTAAGGCCCGAGCAA
Mmp9	CCTGGAACTCACACGACATCTTC	TGGAAACTCACACGCCAGAA
Myh6	AACCAGAGTTTGAGTTTGAGTGACAGAATG	ACTCCGTGCGGATGTCAAA
Myh7	GCGACTCAAAAAGAAGGACTTTG	GGCTTGCTCATCCTCAATCC
Nppb	GCCAGTCTCCAGAGCAATTCA	GGGCCATTTCCTCCGACTT
Nqo1	TATCCTTCCGAGTCATCTCTAGCA	TCTGCAGCTTCCAGCTTCTTG
Parp1	CCGCTTTCACTTCCTCCATCTTC	CTCTCCCAGAACAAGGACGAAG
Pck1	AGCATTCAACGCCAGGTTC	CGAGTCTGTCAGTTCAATACCAA
Pdk4	CACCACATGCTCTTCGAACTCT	AAGGAAGGACGGTTTTCTTGA

Ppara	CAAGGCCTCAGGGTACCACTAC	GCCGAATAGTTCGCCGAAA
Ppard	GAGGAAGTGGCCATGGGTGAC	CCGCCTGAGGCCCCATCACAG
Ppargc1a	AACCACACCCACAGGATCAGA	TCTTCGCTTTATTGCTCCATGA
Serpin1	TCAGCCCTTGCTTGCCTCAT	GCATAGCCAGCACCCAGGA
Slc2a4	AGAGTCTAAAGCGCCT	CCGAGACCAACGTGAA
Smad7	TCTCAAACCAACTGCAGGCT	GGGCCAGATAATTCGTTCCC
Sod2	CAGGACCCATTGCAAGGAA	GTGCTCCCACACGTCAATCC
Tgfb1	GCTCTTGTGACAGCAAAGATAACAA	GGTCGCCCCGACGTTT
Tnf-α	ATGGCCCAGACCCTCACA	TTGCTACGACTGGGCTACA
**Human**		
ATF3	AAGAACGAGAAGCAGCATTTGAT	TTCTGAGCCCGGACAATACAC
BCL2	CTGGGATGCCTTTGTGGAA	CAGCCAGGAGAAATCAAATCAAACAGA
BCL2L1	GCCACAGCAGCATTTGGAT	TCCCTCAGCGCTTGCTTTAC
NPPB	GAGGGCAGGTGGGAAGCAAAC	GCAAGAAGAGCAGGAGCAG-GAG
CCL2	GCTGTGATCTTCAAGACCATTGTG	TGGAATCCTGAACCCACTTCTG
CCn2	CAAGGGCCTCTTCTGTGACT	ACGTGCACTGGTACTTGCAG
COL1A1	GAACGCGTGTCATCCCTTG	GAACGAGGTAGTCTTTCAGCAACA
GAPDH	GGCCTCCAAGGAGTAAGACC	AGGGGTCTACATGGCAACTG
GADD45A	CTTGGAGACCGACGCTGG	TGTAGCGACTTTCCCGGC
IL6	CCCCCAGGAGAAGATTCCAA	TCAATTCGTTCTGAAGAGGTGAGT
MMP2	TTGATGGCATCGCTCAGATC	TGTCACGTGGCGTCACAGT
MMP9	CCACCACAACATCACCTATTGG	GAGGCGCGGGCAAA
TBP	ATGTTGAGTTGCAGGGTGTG	CCCAGATAGCAGCACGGTAT
TGFB1	AACCCACAACGAAATCTATGAC	GAGGTATCGCCAGGAATTG
TNF-α	TCTTCTCGAACCCCGAGTGA	GGAGCTGCCCCTCAGCTT

Supplementary Table 2 List of antibodies used in this study.

Protein	Manufacturer	Cat. number
β-Actin	Sigma-Aldrich Corporation	A5441
ATF3	Merck Millipore	MABN124
ATF4	Santa Cruz Biotechnology	sc-390063
AMPKα	Cell Signaling Technology	#2532
Phospho-AMPKα^{Thr172}	Cell Signaling Technology	#2531
AKT	Cell Signaling Technology	#9272
Phospho-AKT^{Ser473}	Cell Signaling Technology	#9271
BAX	Cell Signaling Technology	#2772
BCL2	Cell Signaling Technology	#2870
c-Caspase 3	Santa Cruz Biotechnology	sc-56053
CHOP (GADD153/DDIT3)	GeneTex Inc.	GTX112827
ERK1/2 (p44/42) MAPK	Cell Signaling Technology	#9102
FOS (c-Fos)	Cell Signaling Technology	#2250
Phospho-ERK1/2$^{Thr202/Tyr204}$ (p44/42) MAPK	Cell Signaling Technology	#9101
JNK/SAPK	Cell Signaling Technology	#9252
Phospho-JNK/SAPK$^{Thr183/Tyr185}$	Cell Signaling Technology	#9251
JUN (c-Jun)	Santa Cruz Biotechnology	sc-44
p65 (NF-κB)	Santa Cruz Biotechnology	sc-109
p38 MAPK	Cell Signaling Technology	#9212
Phospho-p38 MAPK$^{Thr180/Tyr182}$	Cell Signaling Technology	#9211
PARP1	Cell Signaling Technology	#9542
SMAD3	Cell Signaling Technology	#9513
Phospho-SMAD3$^{Ser423/425}$	Cell Signaling Technology	#9520
STAT3	Santa Cruz Biotechnology	sc-482
Phospho-STAT3^{Ser727}	Cell Signaling Technology	#9134
Vinculin	Santa Cruz Biotechnology	sc-73614

Supplementary Table 3 Patients' demographic and clinical characteristics, and pharmacological treatments.

**Variable**[a]	**Aortic stenosis**	**Surgical controls**
**Age (yrs)**	69.3 ± 11.7	52.8 ± 13.7
**Female (%)**	46	56
**Systolic blood pressure (mm Hg)**	122 ± 19	119 ± 18
**Diastolic blood pressure (mm Hg)**	69 ± 12	68 ± 10
**Body Mass Index (kg/m^2)**	29.1 ± 4.6	27.9 ± 4.6
**Body Mass Index ≥ 30 (%)**	35	28
**Current smoker (%)**	13	18
**Previous smoker (%)**	14	14
**Systemic hypertension (%)**	60	44
**Diabetes Mellitus (%)**	21	6
**Hyperlipidemia (%)**	42	20
**Atrial fibrillation or flutter (%)**	15	16
**LV EF**[b] **(%)**	60 ± 12	61 ± 8
**LVMI**[b] **($g/m^{2.7}$)**	73 ± 20	44 ± 20
**ACE**[b] **inhibitors (%)**	25	16
**ATII**[b] **receptor antagonists (%)**	19	16
**Diuretics (%)**	43	24
**Calcium channel blockers (%)**	14	12
**β-Blockers (%)**	18	30
**Statins (%)**	39	4

[a]Data are presented as percentage or mean ± standard deviation (SD).
[b]ACE = Angiotensin converting enzyme; ATII = Angiotensin II; LVEF = left ventricular ejection fraction; LVMI = left ventricular mass index in grams to the 2.7th power of height in meters.

Supplementary Figures: Figure S1

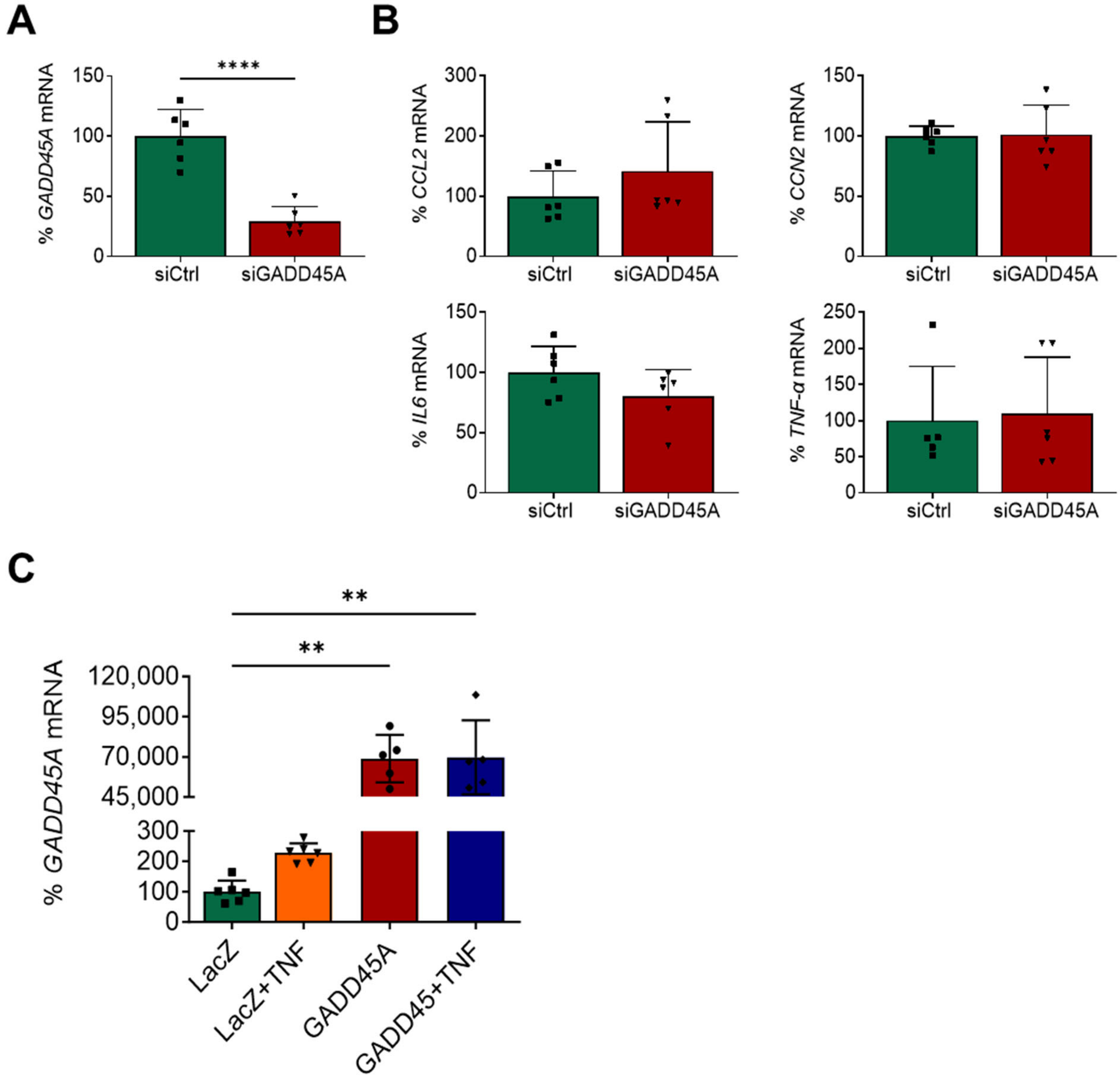

Supplementary Figure S1 Relative quantification of the mRNA expression of *GADD45A* (**A**) and *CCL2*, *CCN2*, *IL6*, and *TNF-α* (**B**) in human AC16 cardiac cells transfected with scrambled siRNA (siRNA control, siCtrl) or GADD45A siRNA (siGADD45A). (**C**) Relative quantification of the mRNA expression of *GADD45A* in human AC16 cardiac cells transfected with LacZ-carrying or GADD45A-carrying plasmids in the presence or absence of TNF-α (TNF, 10 ng/mL, 24 h). The graphs represent the quantification of the glyceraldehyde-3-phosphate dehydrogenase (*GAPDH*)-normalized mRNA levels, expressed as a percentage of the control (A, siCtrl; B, LacZ) samples. Data are presented as the mean ± SD. $*p < 0.05$, $**p < 0.01$, $***p < 0.001$.

Supplementary Figures: Figure S2

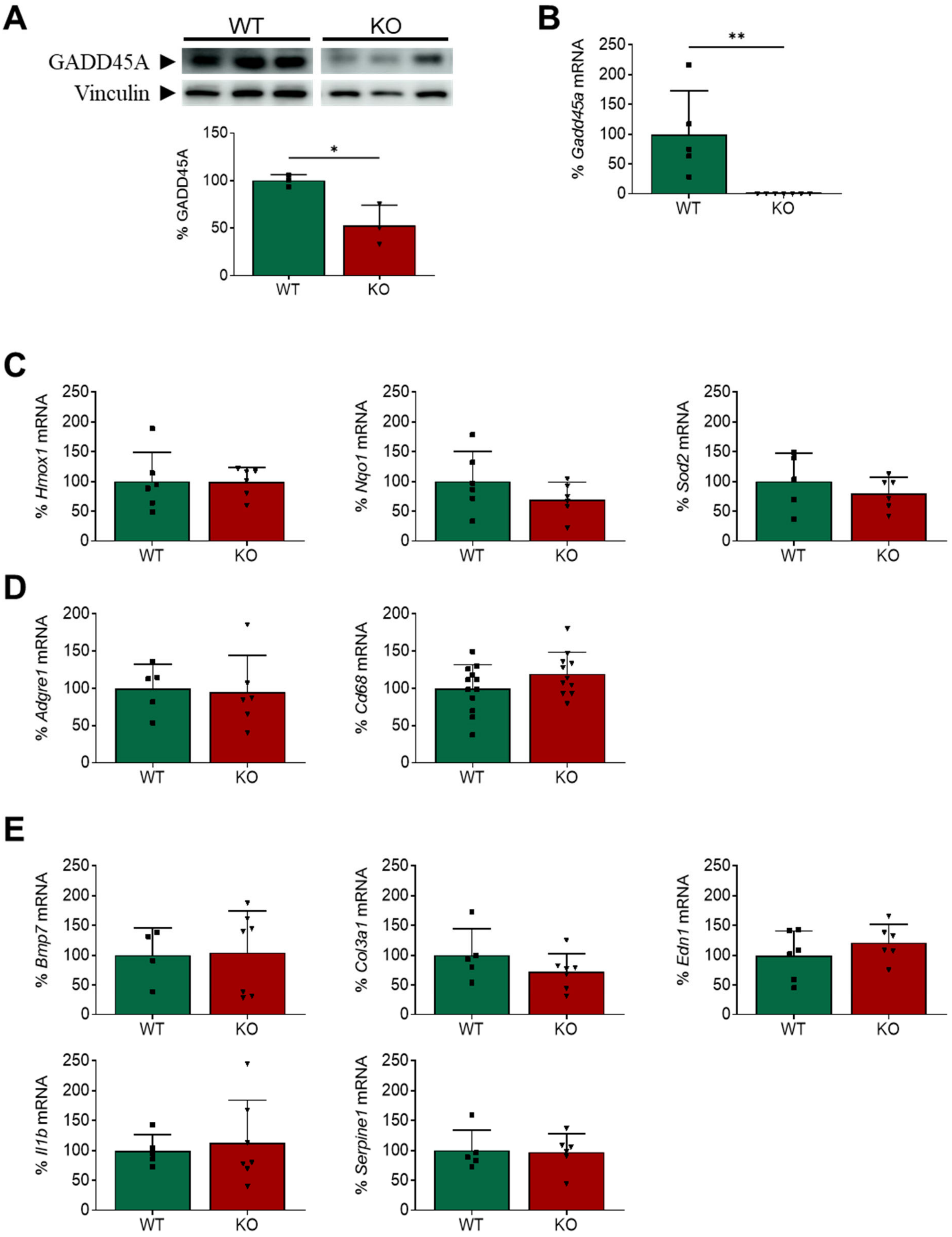

Supplementary Figure S2 (**A**) Western blot analysis showing the protein levels of GADD45A in wild-type (WT) and *Gadd45a* knockout (KO) mice. The graphs represent the quantification of the protein levels normalized to vinculin, and are expressed as a percentage of control samples. Relative quantification of the

mRNA expression of *Gadd45a* (**B**), *Hmox1*, *Nqo1*, *Sod2* (**C**), *Adgre1*, *Cd68* (**D**), and *Bmp7*, *Col3a1*, *Edn1*, *Il1b*, and *Serpine1* (**E**) in the same mice. The mRNA levels were normalized to adenine phosphoribosyl transferase (*Aprt*), and are expressed as a percentage of the control samples. Data are presented as the mean ± SD. *$p < 0.05$, **$p < 0.01$, *** $p < 0.001$

Supplementary Figures: Figure S3

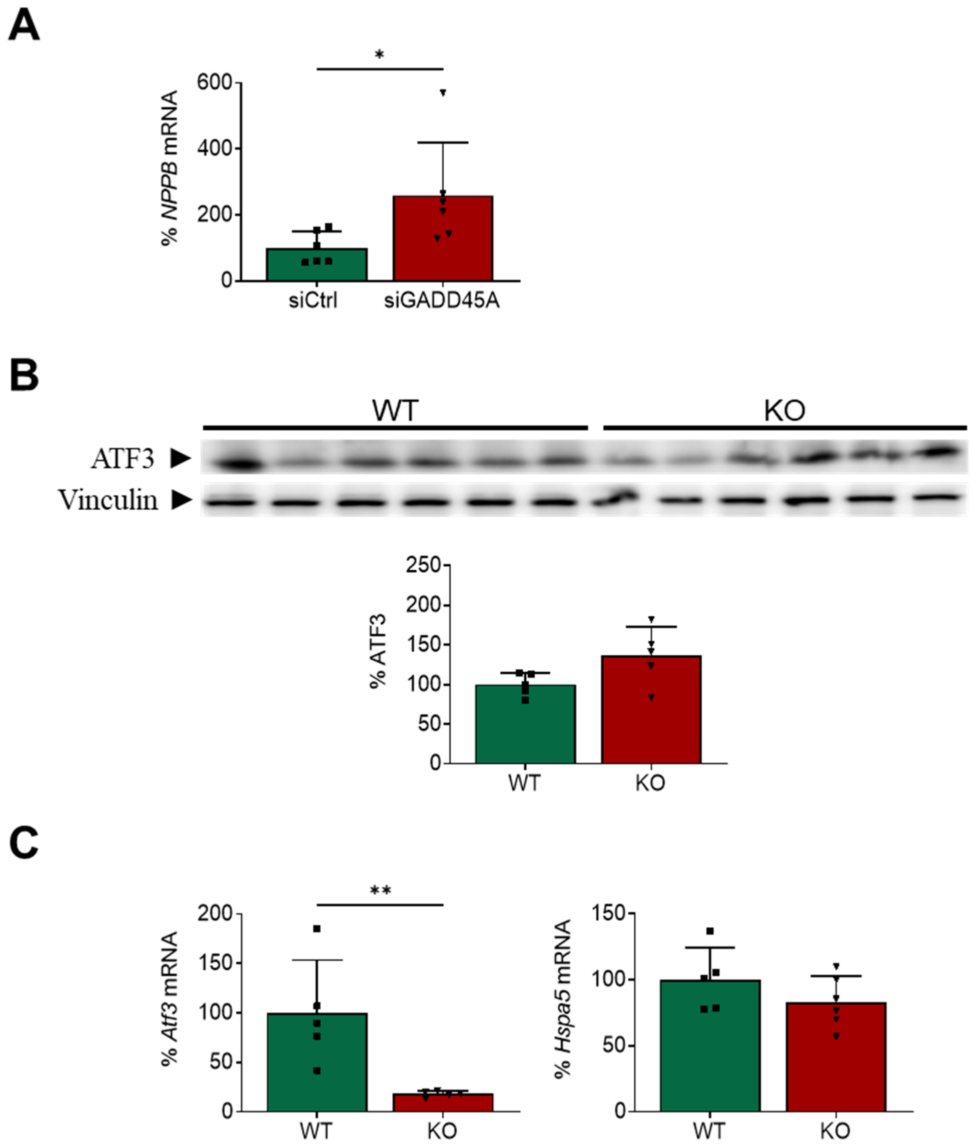

Supplementary Figure S3 (**A**) Relative quantification of the mRNA expression of *NPPB* in human AC16 cardiac cells transfected with scrambled siRNA (siRNA control, siCtrl) or GADD45A siRNA (siGADD45A). The graph represents the quantification of the glyceraldehyde-3-phosphate dehydrogenase (*GAPDH*)-normalized mRNA levels, expressed as a percentage of the control samples. (**B**) Western blot analysis showing the protein levels of ATF3 in wild-type (WT) and *Gadd45a* knockout (KO) mice. The graph represents the quantification of the protein levels normalized to vinculin, and is expressed as a percentage of the control samples. (**C**) Relative quantification of the mRNA expression of *Atf3* and *Hspa5* in wild-type (WT) and *Gadd45a* knockout (KO) mice. The mRNA levels were normalized to adenine phosphoribosyl transferase (*Aprt*), and are expressed as a percentage of the control samples. Data are presented as the mean ± SD. *$p < 0.05$, **$p < 0.01$, *** $p < 0.001$
