# Supplementary material for: GADD45A suppression contributes to cardiac remodeling by promoting inflammation, fibrosis and hypertrophy
Source: Cell Mol Life Sci. 2025 Apr 30;82(1):189. doi: 10.1007/s00018-025-05704-x (PMC12040809; doi:10.1007/s00018-025-05704-x)

SUPPLEMENTARY FILE 2 to:

GADD45A contributes to cardiac remodeling by promoting inflammation, fibrosis and hypertrophy

Adel Rostami, Xavier Palomer, Javier Pizarro-Delgado, Lucía Peña, Mònica Zamora, Marta Montori-Grau, Emma Barroso, Brenda Valenzuela-Alcaraz, Fàtima Crispi, Jesús M. Salvador, Raquel García, María A. Hurlé, Francisco Nistal, Manuel Vázquez-Carrera

Correspondence to: Xavier Palomer (xpalomer@ub.edu; ORCID: 0000-0001-7647-9984) and Manuel Vázquez-Carrera (mvazquezcarrera@ub.edu; ORCID: 0000-0001-7138-8207), Department of Pharmacology, Toxicology and Therapeutic Chemistry, Faculty of Pharmacy and Food Sciences, University of Barcelona, Av. Joan XXIII 27-31, E-08028, Barcelona, Spain. Tel: +34 934024531.

Figure 4B

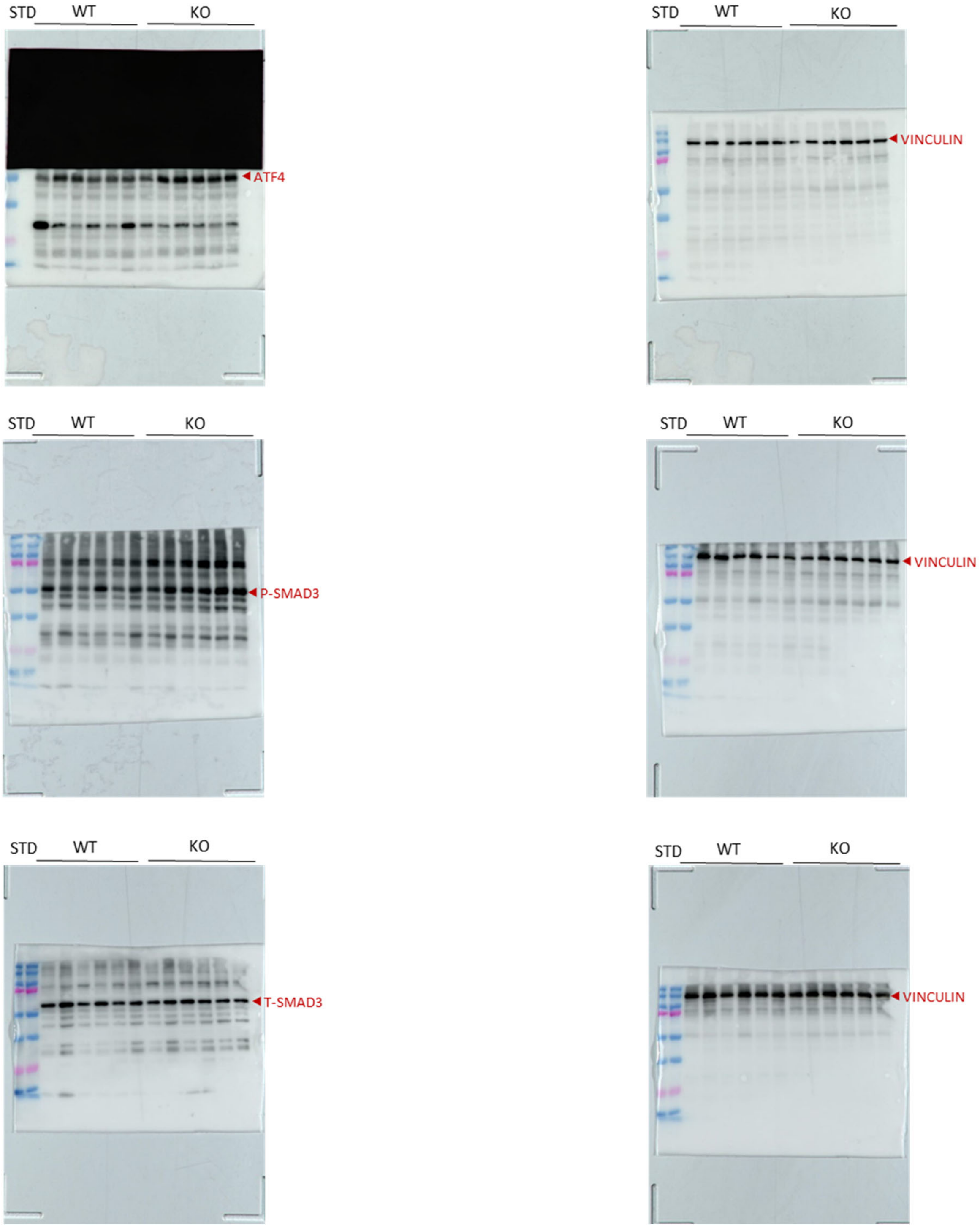
STD
WT
KO
ATF4
STD
WT
KO
VINCULIN
STD
WT
KO
P-SMAD3
STD
WT
KO
VINCULIN
STD
WT
KO
T-SMAD3
STD
WT
KO
VINCULIN

Figure 4E (1/2)

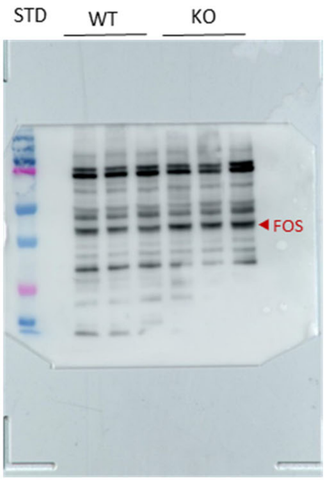
STD
WT
KO
FOS

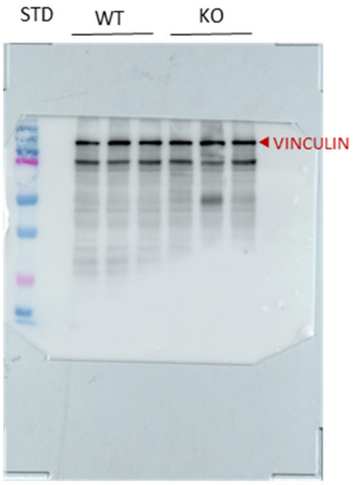
STD
WT
KO
VINCULIN

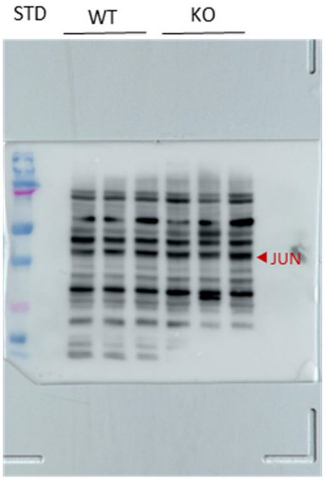
STD
WT
KO
JUN

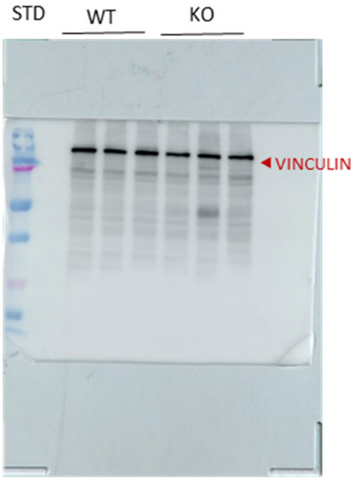
STD
WT
KO
VINCULIN

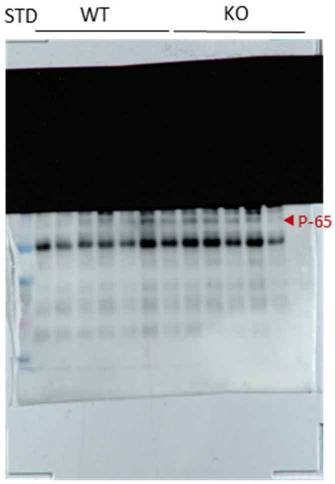
STD
WT
KO
P-65

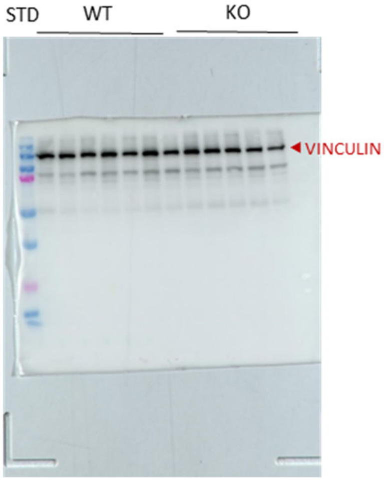
STD
WT
KO
VINCULIN

Figure 4E (2/2)

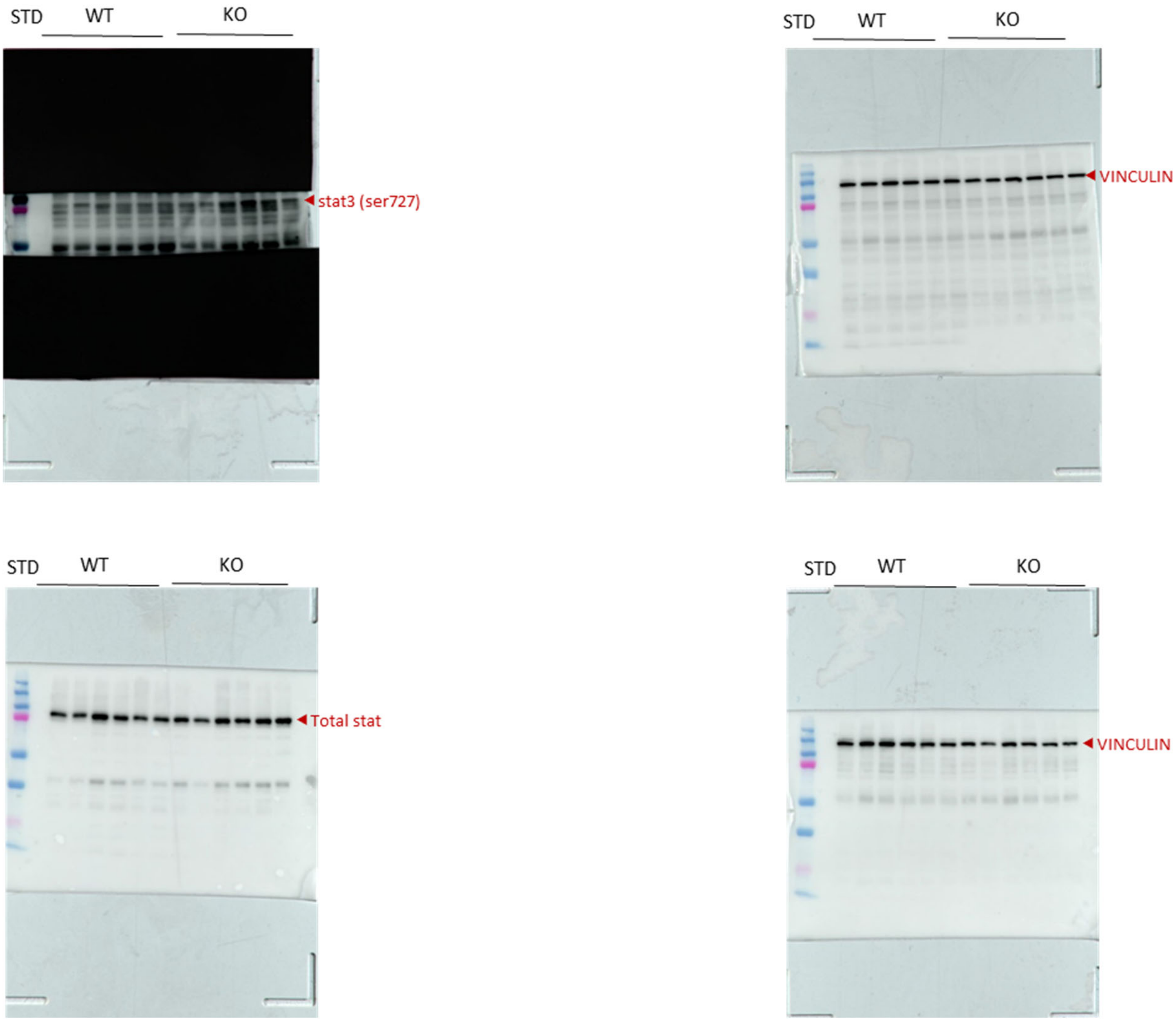
STD
WT
KO
stat3 (ser727)
STD
WT
KO
VINCULIN
STD
WT
KO
Total stat
STD
WT
KO
VINCULIN

Figure 5A

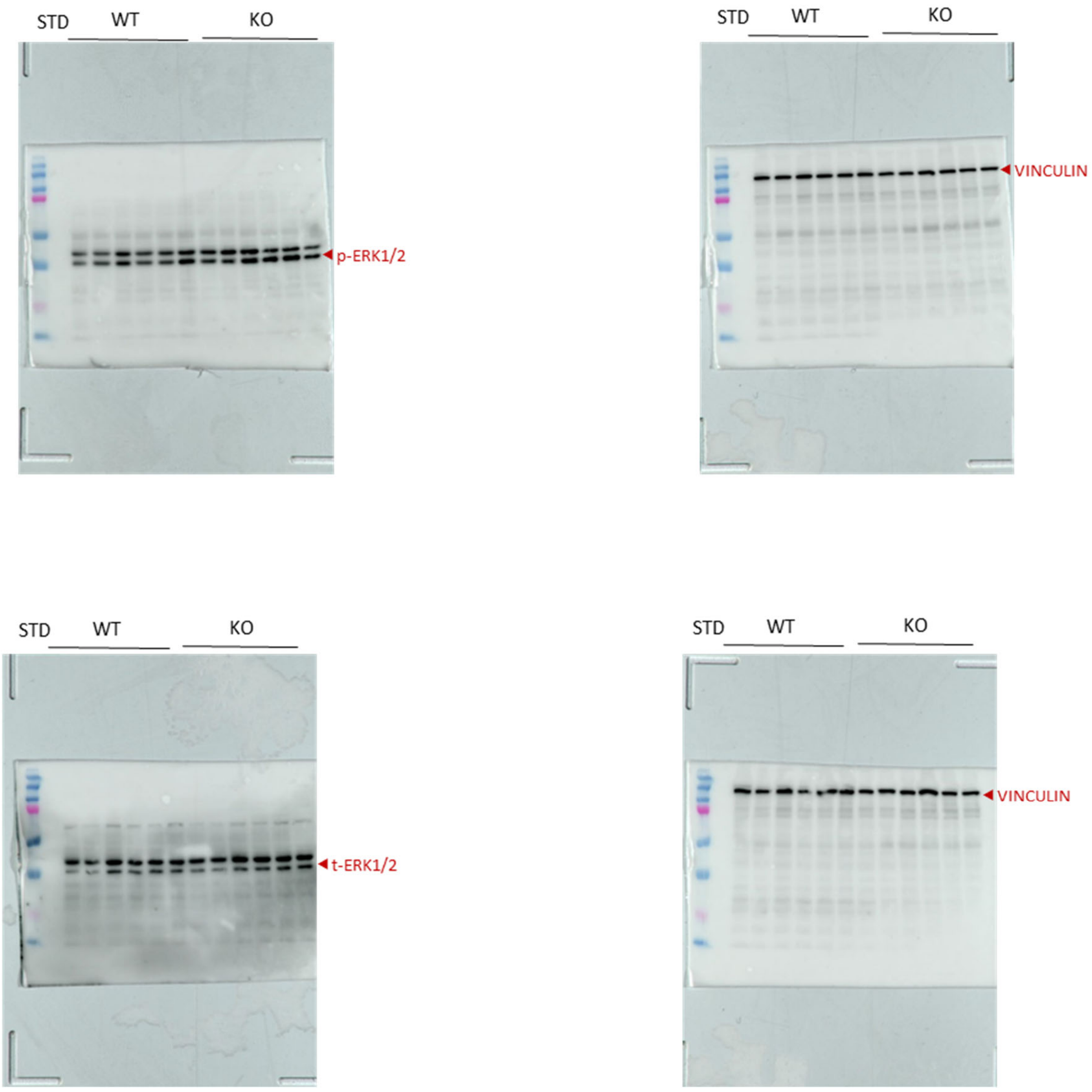
STD
WT
KO
p-ERK1/2
STD
WT
KO
VINCULIN
STD
WT
KO
t-ERK1/2
STD
WT
KO
VINCULIN

Figure 5B

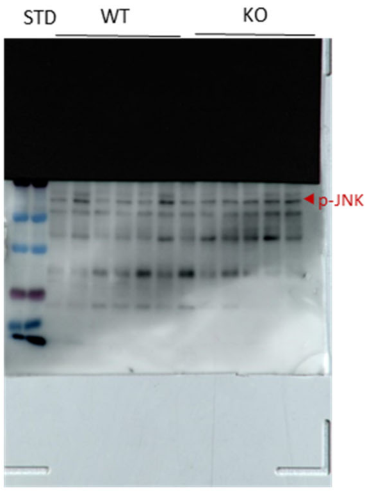
STD
WT
KO
p-JNK

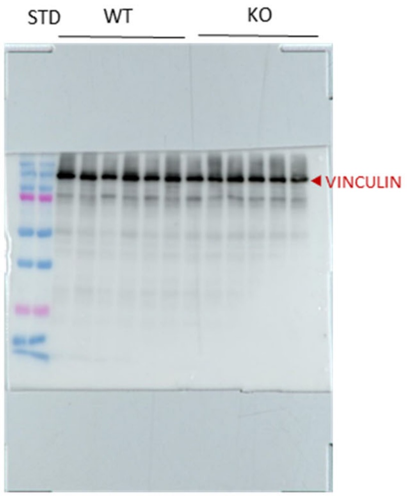
STD
WT
KO
VINCULIN

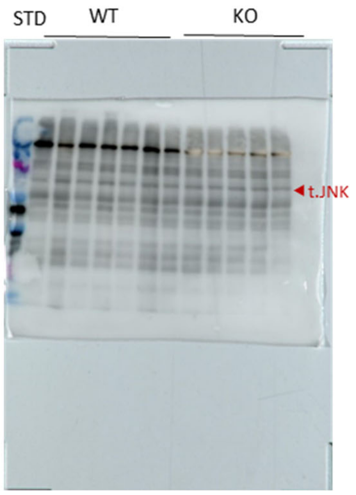
STD
WT
KO
t.JNK

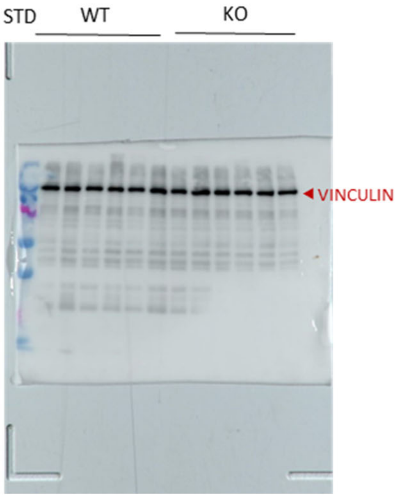
STD
WT
KO
VINCULIN

Figure 5C

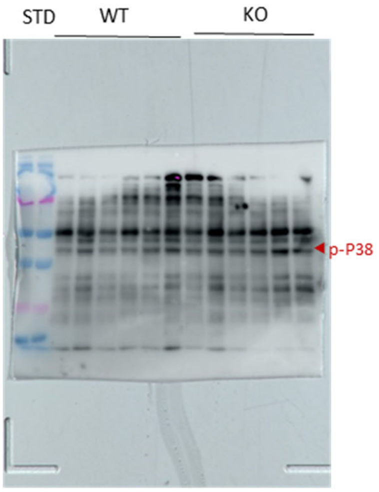
STD
WT
KO
p-P38

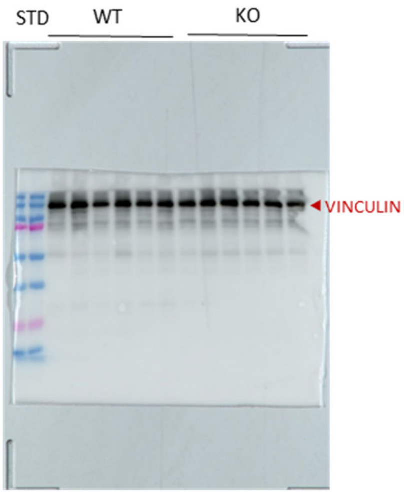
STD
WT
KO
VINCULIN

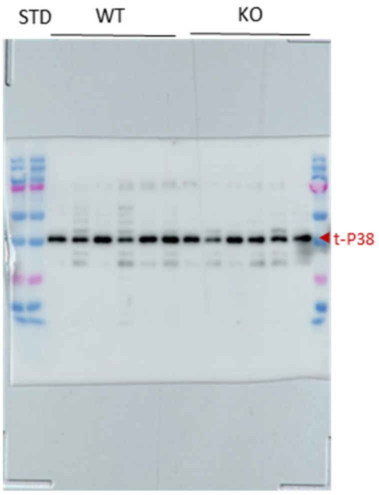
STD
WT
KO
t-P38

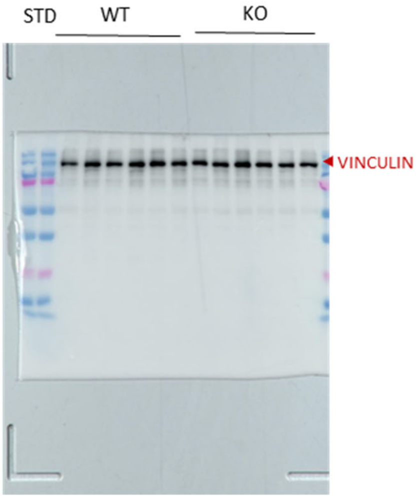
STD
WT
KO
VINCULIN

Figure 7A

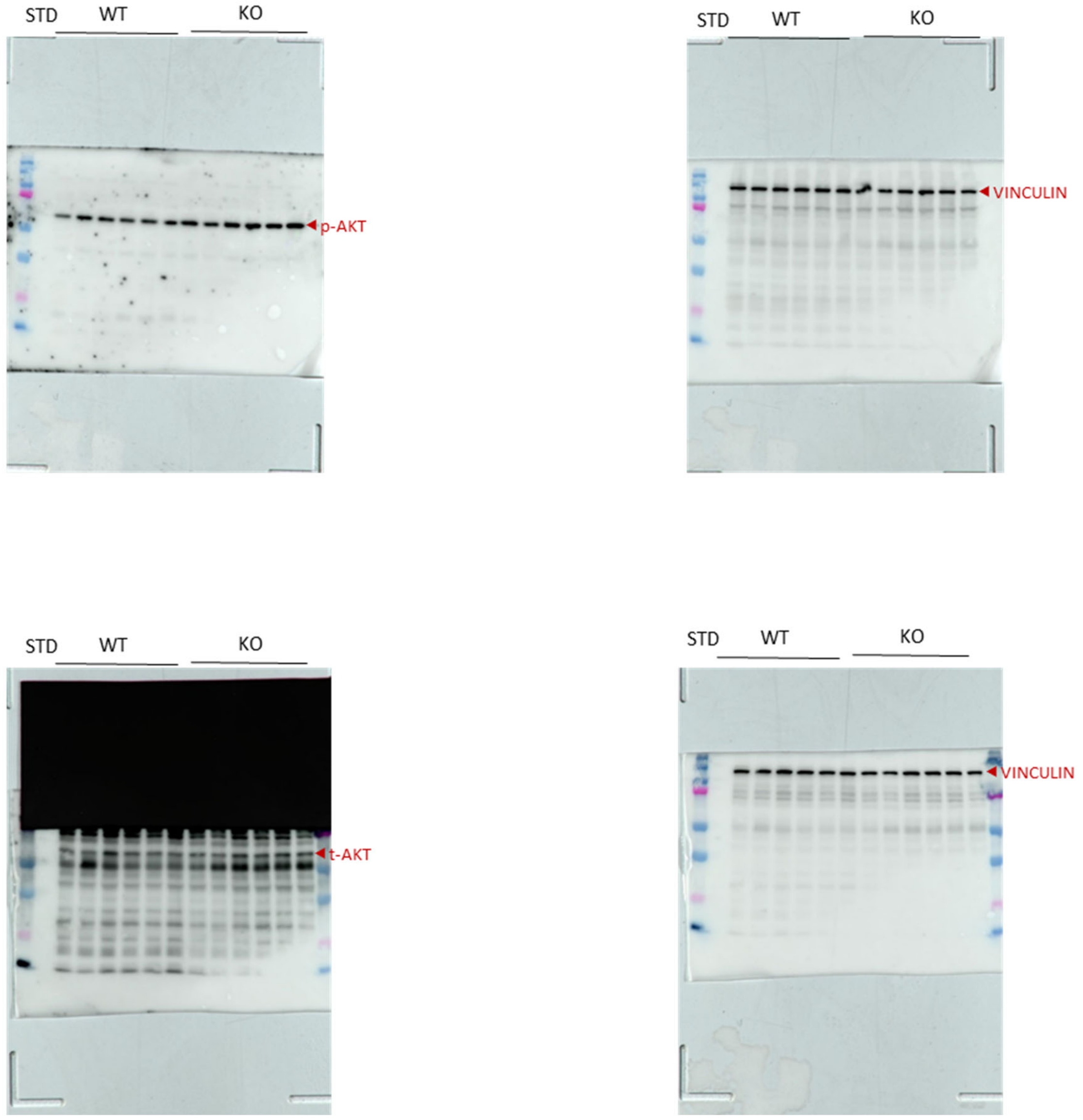
STD
WT
KO
p-AKT
STD
WT
KO
VINCULIN
STD
WT
KO
t-AKT
STD
WT
KO
VINCULIN

Figure 7B

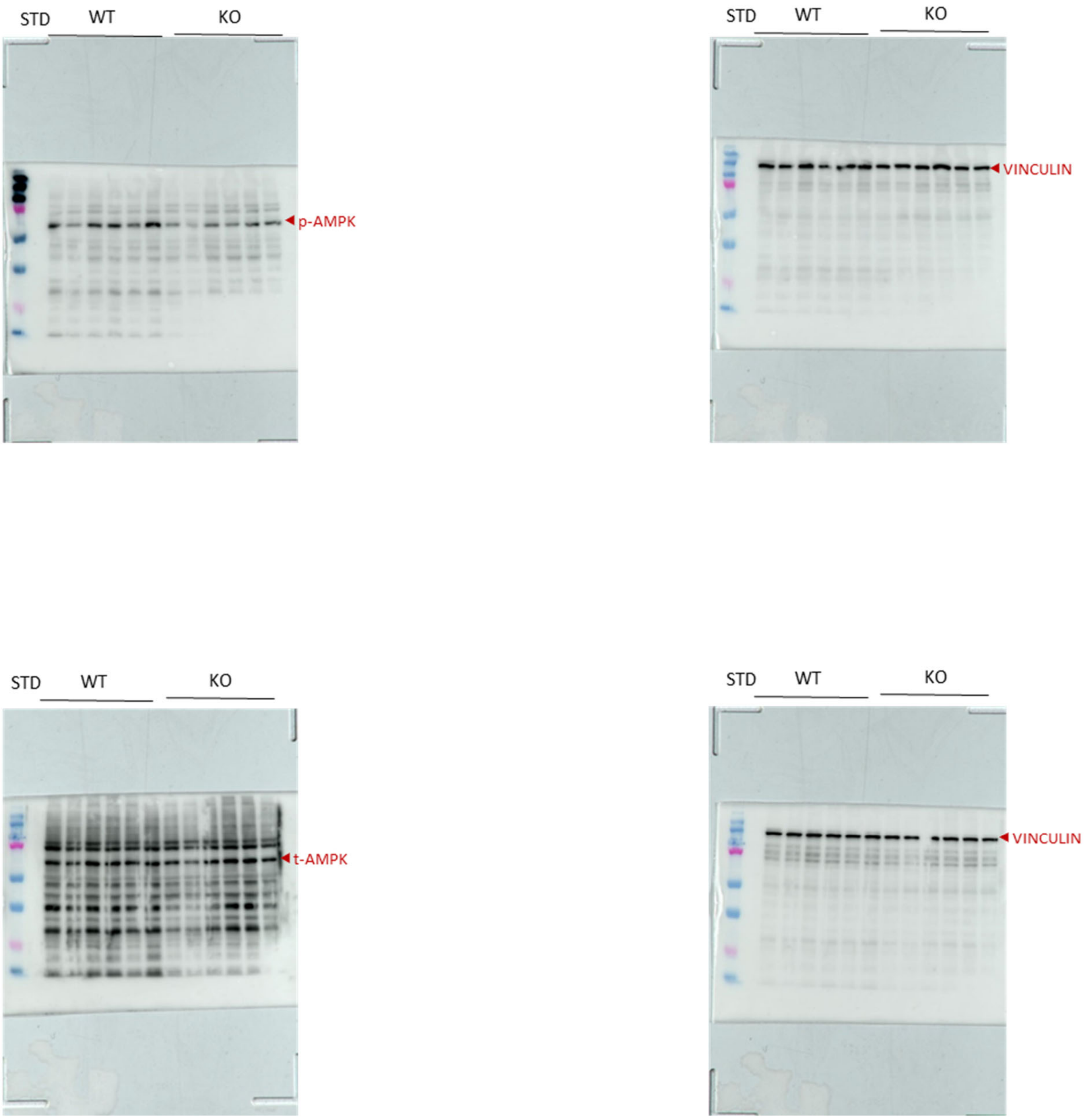
STD
WT
KO
p-AMPK
STD
WT
KO
VINCULIN
STD
WT
KO
t-AMPK
STD
WT
KO
VINCULIN

Figure 7C (1/2)

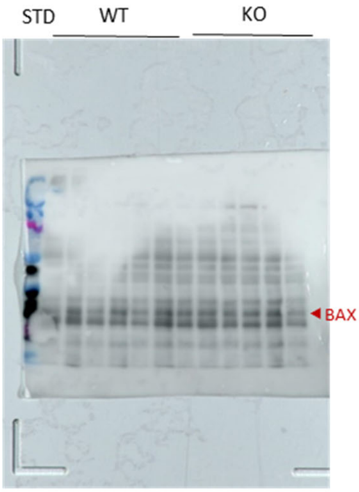
STD
WT
KO
BAX

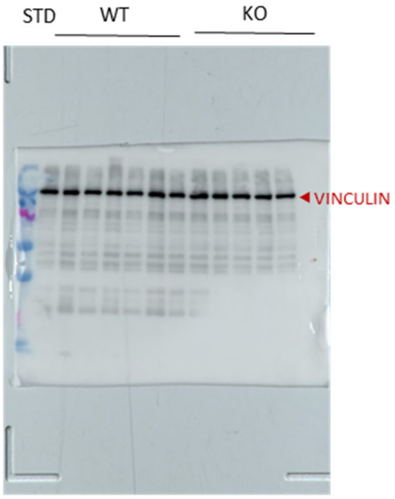
STD
WT
KO
VINCULIN

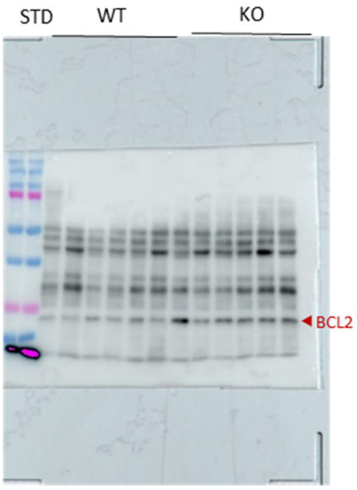
STD
WT
KO
BCL2

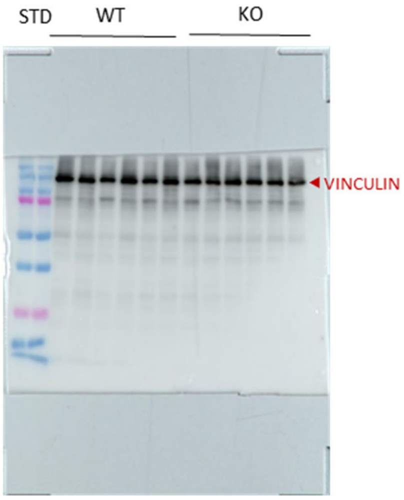
STD
WT
KO
VINCULIN

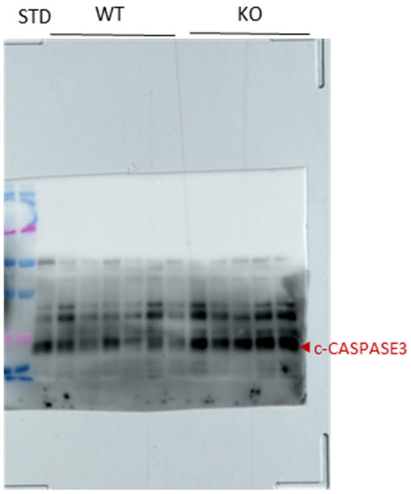
STD
WT
KO
c-CASPASE3

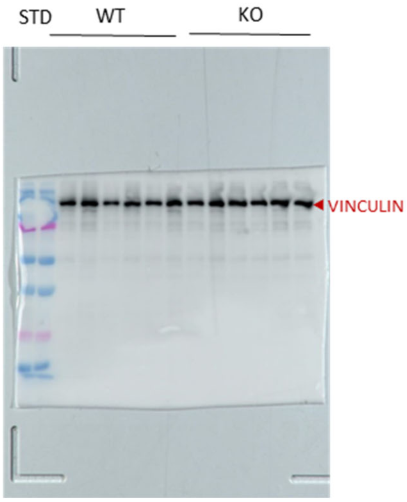
STD
WT
KO
VINCULIN

Figure 7C (2/2)

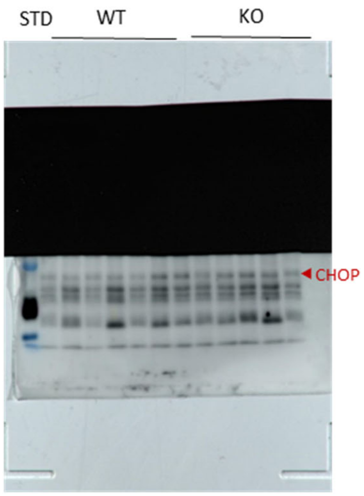
STD
WT
KO
CHOP

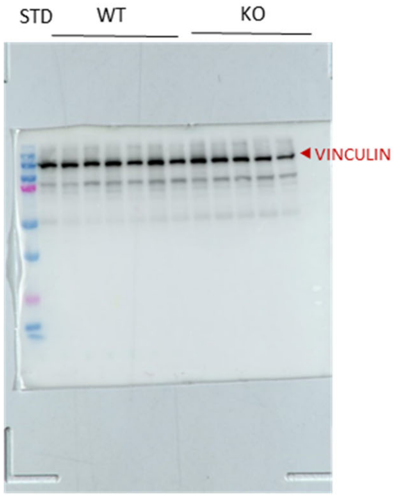
STD
WT
KO
VINCULIN

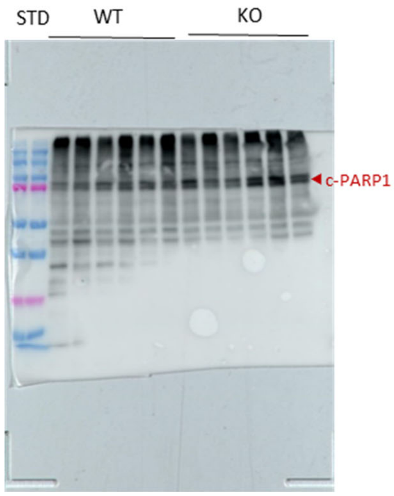
STD
WT
KO
c-PARP1

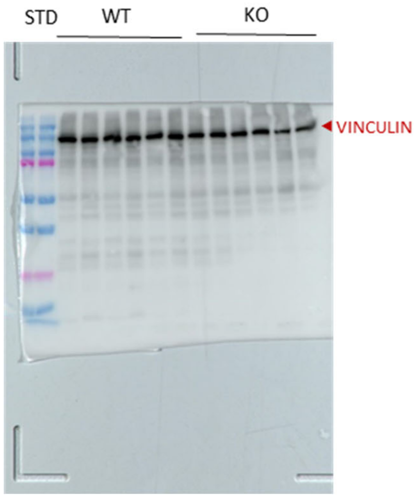
STD
WT
KO
VINCULIN

Supplemental Figure S2A

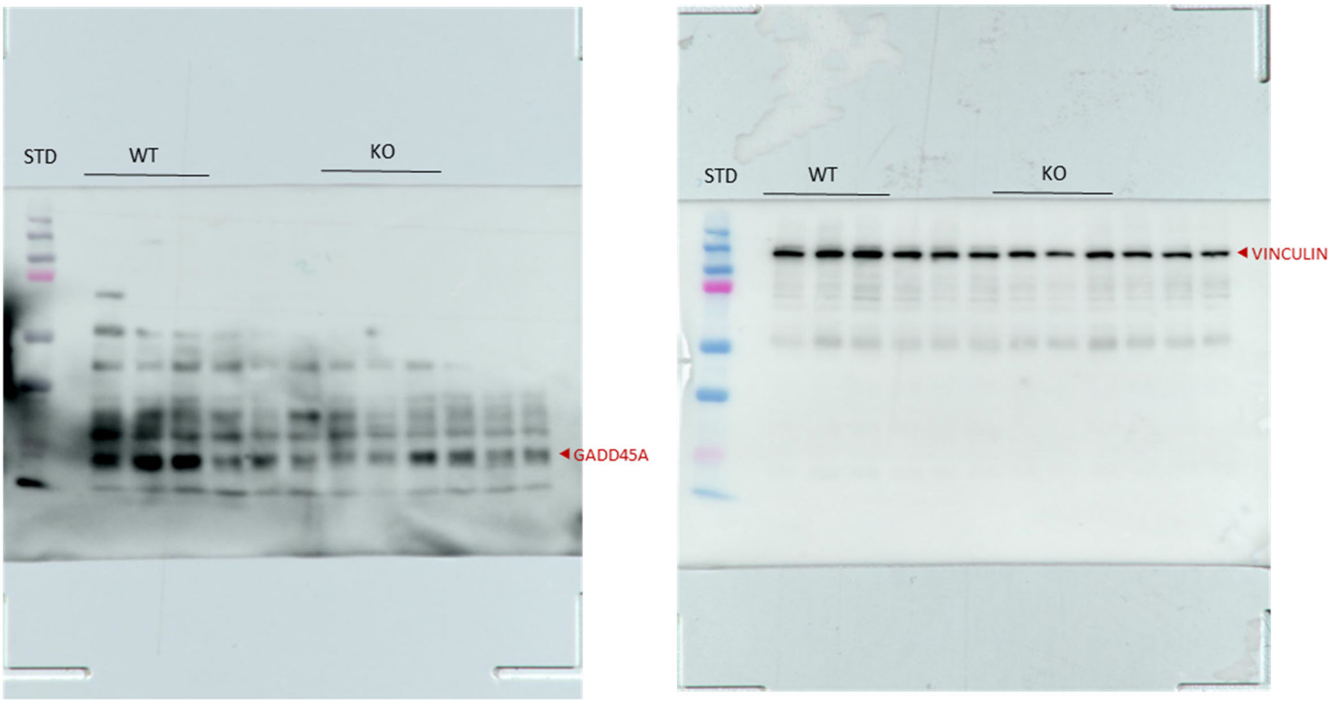

Supplemental Figure S3

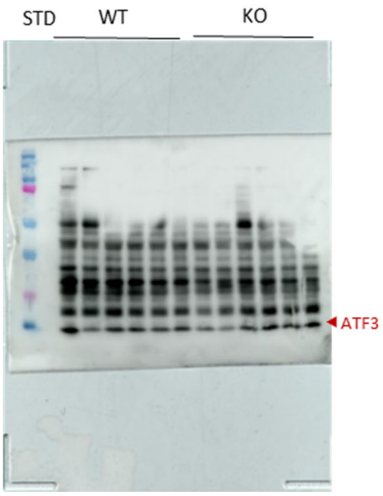

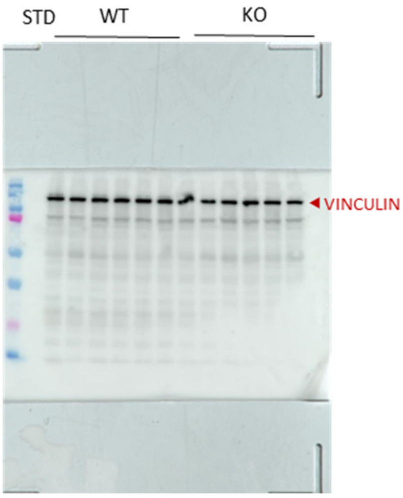

Supplement: Supplementary file 2 — Supplementary Material 2 [file 18_2025_5704_MOESM2_ESM.pdf]
